# The Fisher–Rao Distance between Multivariate Normal Distributions: Special Cases, Bounds and Applications

**DOI:** 10.3390/e22040404

**Published:** 2020-04-01

**Authors:** Julianna Pinele, João E. Strapasson, Sueli I. R. Costa

**Affiliations:** 1 Center of Exact and Technological Sciences, University of Reconcavo of Bahia, Cruz das Almas 44380-000, Brazil; 2 School of Applied Sciences, University of Campinas, Limeira 13484-350, Brazil; joao.strapasson@fca.unicamp.br; 3 Institute of Mathematics, University of Campinas, Campinas 13083-859, Brazil; sueli@ime.unicamp.br

**Keywords:** information geometry, Fisher–Rao distance, multivariate normal distributions, Gaussian mixture simplification

## Abstract

The Fisher–Rao distance is a measure of dissimilarity between probability distributions, which, under certain regularity conditions of the statistical model, is up to a scaling factor the unique Riemannian metric invariant under Markov morphisms. It is related to the Shannon entropy and has been used to enlarge the perspective of analysis in a wide variety of domains such as image processing, radar systems, and morphological classification. Here, we approach this metric considered in the statistical model of normal multivariate probability distributions, for which there is not an explicit expression in general, by gathering known results (closed forms for submanifolds and bounds) and derive expressions for the distance between distributions with the same covariance matrix and between distributions with mirrored covariance matrices. An application of the Fisher–Rao distance to the simplification of Gaussian mixtures using the hierarchical clustering algorithm is also presented.

## 1. Introduction

A proper measure to determine the dissimilarity between probability distributions has been approached in many problems and applications. The Fisher–Rao distance is a very special metric for statistical models of probability distributions. This distance is invariant by reparametrization of the sample space and covariant by reparameterization of the parameter space [1]. Moreover, the Fisher–Rao metric is preserved under Markov morphisms and under centain conditions it is, up to a scaling factor, the unique Riemannian metric satisfying this condition [2,3]. Markov morphisms are associated with the notion of statistical sufficiency which express the criterion of passing from one statistical model to another with no loss of information [4,5,6]. Therefore it is natural to require the invariance of the geometric structures of statistical models under Markov morphisms. Between finite sample size simplex model $S_{k-1}=\left\{p\in R^{k};p_{i}\geq 0\;\mathrm{and}\;\sum_{i=1}^{k}p_{i}=1\right\}$, a Markov morphism is a linear map $T_{Q}\left(x\right)=xQ$, where $Q\in R^{n\times l}$, with n≤l, is a matrix with non-negative entries such that every row sums to 1 and every column has precisely one non-zero element. The mapping $T_{Q}$ corresponds to probabilistic refining of the event space {1,…,n}→{1,…,l} where the refinement i→j occurs with probability $Q_{ij}$ [7]. Chentsov [8,9] has proved the Fisher–Rao uniqueness invariance property under Markov morphisms for the finite sample spaces. The extension of this result to more general statistical models requires careful formulations of statistical sufficiency and Markov morphisms and has been evolved since then [3,10]. More recently in [5,6] it is shown this uniqueness of the Fisher–Rao metric under an assumption of strong continuity of the information metric.

After previous papers [11,12,13] connecting geometry and statistics, C. R.Rao in an independent landmark paper [14] considered statistical models with the metric induced by the information matrix defined by R. Fisher in 1921 [15]. This work encouraged several authors to calculate the Fisher–Rao metric distance between other probability distributions [16,17,18] as well as stimulated approaches to other dissimilarity measures such as Kullback-Leibler divergence [19], total variation and Wasserstein distances [20]. Amari [3,4,21] unified the information geometry theory by organizing and introducing other concepts regarding statistical models [2].

An explicit form for the Fisher–Rao distance in the univariate normal distribution space is known via an association with the classical model of the hyperbolic plane [14,16,18,22]. It was applied to quantization of hyperspectral images [23] and to the space of projected lines in the paracatadioptric images [24]. This Fisher–Rao model was used to simplify Gaussian mixtures through the *k*-means method [25] and a hierarchical clustering technique [26].

An expression for the geodesic curve (initial value problem) in the multivariate normal distributions space was derived in [27] and in [28]. However, the calculus of the Fisher–Rao distance requires solving non-trivial differential equations under boundary conditions to find the geodesic connecting two distributions and then to calculate the integral along the geodesic. A closed form for this distance in the general case is still an open problem. Expressions for the distance are known only in special cases [16,17,18].

The Fisher–Rao distance between multivariate normal distributions in specific cases, such as distributions with a common mean, was considered in diffusion tensor image analysis [29,30,31], in color texture discrimination in several classification experiments [32], in the problem of distributed estimation fusion with unknown correlations [33], and in the machine learning technique [34]. In [35,36], the authors described shapes representing landmarks by a Gaussian model with diagonal covariance matrices and used the Fisher–Rao distance to quantify the difference between two shapes. In [17], this model was applied to statistical inference. Bounds for the Fisher–Rao distance were used to track quality monitoring [37].

This paper is organized as follows. In Section 2, we gather known results (closed forms for special cases and bounds) for the Fisher–Rao distance between multivariate normal distributions. In Section 3, we describe a closed form for the Fisher–Rao distance between distributions with the same covariance matrix and a non-linear system to find the distance between distributions with mirrored covariance matrices. An application of the Fisher–Rao distance to the simplification of Gaussian mixtures using the hierarchical clustering algorithm is presented in Section 4. Some conclusions and perspectives are drawn in Section 5.

## 2. The Fisher–Rao Distance in the Multivariate Normal Distribution Space: Special Submanifolds and Bounds

In this section, as in [38], we summarize previous results regarding the Fisher–Rao distance in the space of multivariate normal distributions including closed forms for this distance restricted to submanifolds and general bounds.

Given a statistical model $S=\{p_{\theta }=p\left(x;\theta \right);\theta =\left(\theta_{1},\theta_{2},\ldots ,\theta_{k}\right)\in \Theta \subset R^{k}\}$, a natural Riemannian structure [21] can be provided by the Fisher information matrix $G\left(\theta \right)=\left[g_{ij}\left(\theta \right)\right]$:

(1) $$\begin{array}{cc} g_{ij}\left(\theta \right)= & E_{\theta }\left(\frac{\partial }{\partial \theta_{i}}\log p\left(x;\theta \right)\frac{\partial }{\partial \theta_{j}}\log p\left(x;\theta \right)\right) \end{array}$$

(2) $$\begin{array}{cc} = & \int \frac{\partial }{\partial \theta_{i}}\log p\left(x;\theta \right)\frac{\partial }{\partial \theta_{j}}\log p\left(x;\theta \right)p\left(x;\theta \right)dx, \end{array}$$

where $E_{\theta }$ is the expected value with respect to the distribution $p_{\theta }$. This matrix can also be viewed as the Hessian matrix of the Shannon entropy (concave function) [39],

(3) H(p)=−∫p(x;θ)logp(x;θ)dx,

and is used to establish connections between inequalities in information theory and geometrical inequalities.

The Fisher–Rao distance, $d_{F}\left(\cdot ,\cdot \right)$, between two distributions $p_{\theta_{1}}$ and $p_{\theta_{2}}$ in S, identified with their parameters $\theta_{1}$ and $\theta_{2}$, is given by the shortest length of a curve γ(t) in the parameter space Θ connecting these distributions, $d_{F}\left(p_{\theta_{1}},p_{\theta_{2}}\right)\equiv d_{F}\left(\theta_{1},\theta_{2}\right)=\min_{\gamma }\int {\left|\gamma^{\prime }\left(t\right)\right|}_{G}dt$, where $|\gamma^{\prime }{\left(t\right)|}_{G}=\sqrt{\gamma^{\prime }{\left(t\right)}^{t}G\left(\theta \right)\gamma \left(t\right)}$. Note that this is in fact a metric, since for any $\theta_{1}$, $\theta_{2}$, and $\theta_{3}$ in Θ, we have: (i) $d_{F}\left(\theta_{1},\theta_{2}\right)\geq 0$ and $d_{F}\left(\theta_{1},\theta_{2}\right)=0$ if only if $\theta_{1}=\theta_{2}$; (ii) $d_{F}\left(\theta_{1},\theta_{2}\right)=d_{F}\left(\theta_{2},\theta_{1}\right)$; (iii) $d_{F}\left(\theta_{1},\theta_{2}\right)\leq d_{F}\left(\theta_{1},\theta_{3}\right)+d_{F}\left(\theta_{3},\theta_{2}\right)$. A curve that provides the shortest length is called a geodesic and is given by the solutions of the differential equations

(4) $$\frac{d^{2}\theta_{m}}{dt^{2}}+\sum_{i,j}\Gamma_{ij}^{m}\frac{d\theta_{i}}{dt}\frac{d\theta_{j}}{dt}=0,\;m=1,\cdots ,k,$$

where $\Gamma_{ij}^{m}$ are the Christoffel symbols,

(5) $$\Gamma_{ij}^{m}=\frac{1}{2}\sum_{l}\left(\frac{\partial g_{jl}}{\partial \theta_{i}}+\frac{\partial g_{li}}{\partial \theta_{j}}-\frac{\partial g_{ij}}{\partial \theta_{l}}\right)g^{lm}$$

and $\left[g^{ij}\right]$ is the inverse matrix of the Fisher information matrix.

We consider here the space of the multivariate normal distributions given by:

(6) $$p\left(x;\mu ,\Sigma \right)=\frac{{\left(2\pi \right)}^{-\left(\frac{n}{2}\right)}}{\sqrt{Det(\Sigma )}}\exp\left(-\frac{{\left(x-\mu \right)}^{t}\Sigma^{-1}\left(x-\mu \right)}{2}\right),$$

where $x^{t}=\left(x_{1},\ldots ,x_{n}\right)\in R^{n}$ is the variable vector, $\mu^{t}=\left(\mu_{1},\ldots ,\mu_{n}\right)\in R^{n}$ is the mean vector, and Σ is the covariance matrix in $P_{n}\left(R\right)$, the space of order *n* positive definite symmetric matrices.

In this case, the model $S=M=\{p_{\theta };\theta =\left(\mu ,\Sigma \right)\in R^{n}\times P_{n}\left(R\right)\}$ is a statistical $\left(n+\frac{n(n+1)}{2}\right)$-dimensional manifold.

In this case, the model $S=M=\{p_{\theta };\theta =\left(\mu ,\Sigma \right)\in R^{n}\times P_{n}\left(R\right)\}$ is a statistical manifold of dimension $k=\left(n+\frac{n(n+1)}{2}\right)$. Considering a parametrization $\left(\mu ,\Sigma \right)=\phi \left(\theta_{1},\ldots ,\theta_{k}\right)$ of the model M, the Fisher information matrix is given by [40]

(7) $$g_{ij}\left(\theta \right)=\frac{\partial \mu^{t}}{\partial \theta_{i}}\Sigma^{-1}\frac{\partial \mu }{\partial \theta_{j}}+\frac{1}{2}\mathrm{tr}\left(\Sigma^{-1}\frac{\partial \Sigma }{\partial \theta_{i}}\Sigma^{-1}\frac{\partial \Sigma }{\partial \theta_{i}}\right).$$

The metric provided by this matrix is invariant with respect to affine transformations. In other words, for any $\left(c,Q\right)\in R^{n}\times GL_{n}\left(R\right)$, where $GL_{n}\left(R\right)$ is the group of non-singular *n*-square matrices, the mapping:

(8) $$\begin{array}{cccc} \psi_{\left(c,Q\right)}:\; & M & \to & M\\ \; & \left(\mu ,\Sigma \right) & \mapsto & (Q\mu +c,Q\Sigma Q^{t}), \end{array}$$

is an isometry in M [16]. Consequently, the Fisher–Rao distance between $\theta_{1}=\left(\mu_{1},\Sigma_{1}\right)$ and $\theta_{2}=\left(\mu_{2},\Sigma_{2}\right)$ in M satisfies:

(9) $$d_{F}\left(\theta_{1},\theta_{2}\right)=d_{F}\left(\left(Q\mu_{1}+c,Q\Sigma_{1}Q^{t}\right),\left(Q\mu_{2}+c,Q\Sigma_{2}Q^{t}\right)\right)$$

for any $\left(c,Q\right)\in R^{n}\times GL_{n}\left(R\right)$. In particular, for $Q=\Sigma_{1}^{-(1/2)}$ and $c=-\Sigma_{1}^{\left(-1/2\right)}\mu_{1}$, $\theta_{3}=\left(\mu_{3},\Sigma_{3}\right)=\left(\Sigma_{1}^{-(1/2)}\left(\mu_{2}-\mu_{1}\right),\Sigma_{1}^{-(1/2)}\Sigma_{2}\Sigma_{1}^{-(1/2)}\right)$, the Fisher–Rao distance admits the form:

(10) $$d_{F}\left(\theta_{1},\theta_{2}\right)=d_{F}\left(\theta_{0},\theta_{3}\right),$$

where $\theta_{0}=\left(0,I_{n}\right)$, $I_{n}$ is the *n*-order identity matrix, and $0\in R^{n}$ is the null vector.

The geodesic equations in M can be expressed as [17]:

(11) $$\left\{\begin{array}{c} \frac{d^{2}\mu }{dt^{2}}-\left(\frac{d\Sigma }{dt}\right)\Sigma^{-1}\left(\frac{d\mu }{dt}\right)=0\\ \frac{d^{2}\Sigma }{dt^{2}}+\left(\frac{d\mu }{dt}\right){\left(\frac{d\mu }{dt}\right)}^{t}-\left(\frac{d\Sigma }{dt}\right)\Sigma^{-1}\left(\frac{d\Sigma }{dt}\right)=0. \end{array}\right.$$

and could be partially integrated [27]:

(12) $$\left\{\begin{array}{c} \frac{d\mu }{dt}=\Sigma x\\ \frac{d\Sigma }{dt}=\Sigma \left(B-x^{t}\mu \right), \end{array}\right.$$

(13) $$\left\{\begin{array}{c} \frac{d\Delta }{dt}=-B\Delta +x\delta^{t}\\ \frac{d\delta }{dt}=-B\delta +\left(1+\delta \Delta^{-1}\delta \right)x \end{array}\right..$$

where $\left(\delta \left(t\right),\Delta \left(t\right)\right)=\left(\Sigma^{-1}\left(t\right)\mu \left(t\right),\Sigma^{-1}\left(t\right)\right)$, $x\in R^{n}$, and *B* is a symmetric matrix. The initial conditions for this problem can be taken as:

(14) $$\left\{\begin{array}{c} \left(\delta \left(0\right),\Delta \left(0\right)\right)=\left(0,I_{n}\right)\\ \left(\frac{d\delta }{dt}\left(0\right),\frac{d\Delta }{dt}\left(0\right)\right)=\left(x,-B\right). \end{array}\right.$$

Eriksen [27] and Calvo and Oller [28], in independent works, solved this initial value problem. An explicit solution to the geodesic curve in M [28] is:

(15) $$\left\{\begin{array}{cc} \delta (t)= & -B\left(\cosh\left(tG\right)-I_{n}\right){\left(G^{-}\right)}^{2}x+\sinh\left(tG\right)G^{-}x\\ \Delta (t)= & I_{n}+\frac{1}{2}\left(\cosh\left(tG\right)-I_{n}\right)+\frac{1}{2}B\left(\cosh\left(tG\right)-I_{n}\right){\left(G^{-}\right)}^{2}B\\ \; & -\frac{1}{2}\sinh\left(tG\right)G^{-}B-\frac{1}{2}B\sinh\left(tG\right)G^{-} \end{array}\right.,$$

where $I_{n}$ is an *n*-order identity matrix, $G^{2}=B^{2}+2xx^{t}$, and $G^{-}$ is the generalized inverse square matrix of *G*, that is $GG^{-}G=G$.

Due the fact that the geodesic curve has constant velocity at any point, given (x,B) in the tangent space of M, the Fisher–Rao distance between $\left(0,I_{n}\right)$ and (δ(1),Δ(1)) is:

(16) $$\int_{0}^{1}\sqrt{\frac{d\mu }{dt}\left(0\right)\Sigma^{-1}\left(0\right)\frac{d\mu }{dt}\left(0\right)+\frac{1}{2}\mathrm{tr}\left[{\left(\Sigma^{-1}\left(0\right)\frac{\Sigma }{dt}\left(0\right)\right)}^{2}\right]}\;dt=\sqrt{\frac{1}{2}\mathrm{tr}\left(B^{2}\right)+{\left|x\right|}^{2}},$$

where |·| is the standard Euclidean norm. Note that the above expression provides the Fisher–Rao distance between two distributions only if we can determine the initial value problem from the boundary conditions, which usually is very difficult.

Han and Park in [31] presented a numerical shooting method for computing the minimum geodesic distance between two normal distributions, through parallel transport of a vector field defined along the geodesic curve given in Equation (15).

A closed form for the Fisher–Rao distance between two normal distributions in M is still an important open question. Next, we present closed forms for this distance in some submanifolds of M.

### 2.1. Closed Forms for the Fisher–Rao Distance in Submanifolds of M

In this subsection, we consider submanifolds $M_{*}\subset M$ with the distance induced by the Fisher–Rao metric in M. It is important to remark that, in general, given two distributions $\theta_{1}$ and $\theta_{2}$ in $M_{*}$, the distance between $\theta_{1}$ and $\theta_{2}$ when restricted to a submanifold $M_{*}$ is bigger than the distance between $\theta_{1}$ and $\theta_{2}$ in M, that is $d_{M_{*}}\left(\theta_{1},\theta_{2}\right)\geq d_{M}\left(\theta_{1},\theta_{2}\right)$. This is due to the fact that to get $d_{M_{*}}$, we consider the minimum length of restricted curves, which are the ones contained in the submanifold $M_{*}$. We say that $M_{*}$ is totally geodesic if only if $d_{M_{*}}\left(\theta_{1},\theta_{2}\right)=d_{M}\left(\theta_{1},\theta_{2}\right)$, for any $\theta_{1},\theta_{2}\in M_{*}$, which means that the geodesic in M connecting $\theta_{1}$ and $\theta_{2}$ is contained in $M_{*}$.

#### 2.1.1. The Submanifold $M_{\Sigma }$ Where Σ Is Constant

In the *n*-dimensional manifold composed by multivariate normal distributions with common covariance matrix Σ, $M_{\Sigma }=\left\{p_{\theta };\theta =\left(\mu ,\Sigma \right),\;\Sigma =\Sigma_{0}\in P_{n}\left(R\right)\;\mathrm{constant}\right\}$, the Fisher–Rao distance between two distributions $\theta_{1}=\left(\mu_{1},\Sigma_{0}\right)$ and $\theta_{2}=\left(\mu_{2},\Sigma_{0}\right)$ is [18]:

(17) $$d_{\Sigma }\left(\theta_{1},\theta_{2}\right)=\sqrt{{\left(\mu_{1}-\mu_{2}\right)}^{t}\Sigma_{0}^{-1}\left(\mu_{1}-\mu_{2}\right)}.$$

This distance is equal to the Mahalanobis distance [11], which is equal to the Euclidean distance between the image of $\mu_{1}$ and $\mu_{2}$ under the transformation $\mu \mapsto P^{-1}\mu$, where $\Sigma_{0}=PP^{t}$ is the Cholesky decomposition [18]. This distance was one of the first dissimilarity measures between datasets with some correlation. Note that this submanifold is not totally geodesic, as it can be seen even in the space of univariate normal distributions [22] and in Example 1 in the next section.

A geodesic curve $\gamma_{\Sigma }\left(t\right)$ in $M_{\Sigma }$ connecting $\theta_{1}$ and $\theta_{2}$ can be provided by:

(18) $$\gamma_{\Sigma }\left(t\right)=\left(\left(1-t\right)\mu_{1}-t\mu_{2},\Sigma_{0}\right).$$

#### 2.1.2. The Submanifold $M_{\mu }$ Where μ Is Constant

A totally geodesic submanifold of M is given by $M_{\mu }=\left\{p_{\theta };\theta =\left(\mu ,\Sigma \right),\;\mu =\mu_{0}\in R^{n}\;\mathrm{constant}\right\}$ of dimension $\frac{n(n+1)}{2}$ composed by distributions that have the same mean vector $\mu_{0}$. The Fisher–Rao distance in $M_{\mu }$ was studied by several authors in different contexts [16,18,30,41] and for $\theta_{1}=\left(\mu_{0},\Sigma_{1}\right)$ and $\theta_{2}=\left(\mu_{0},\Sigma_{2}\right)$ is given by:

(19) $$d_{F}\left(\theta_{1},\theta_{2}\right)=\sqrt{\frac{1}{2}\sum_{i=1}^{n}{\left[\log\left(\lambda_{i}\right)\right]}^{2}},$$

where $0<\lambda_{1}\leq \lambda_{2}\leq \cdots \leq \lambda_{n}$ are the eigenvalues of $\Sigma_{1}^{-1/2}\Sigma_{2}\Sigma_{1}^{-1/2}$.

An expression for the geodesic curve connecting these two distributions is [30]:

(20) $$\gamma_{\mu }\left(t\right)=\left(\mu_{0},\Sigma_{1}^{1/2}\exp\left(t\log\left(\Sigma_{1}^{-1/2}\Sigma_{2}\Sigma_{1}^{-1/2}\right)\right)\Sigma_{1}^{1/2}\right).$$

#### 2.1.3. The Submanifold $M_{D}$ Where Σ Is Diagonal

Let $M_{D}=\left\{p_{\theta };\;\theta =\left(\mu ,\Sigma \right),\;\Sigma =\mathrm{diag}\left(\sigma_{1}^{2},\sigma_{2}^{2},\ldots ,\sigma_{n}^{2}\right),\;\sigma_{i}>0,\;i=1,\ldots ,n\right\}$, the submanifold of M composed by distributions with a diagonal covariance matrix. If we consider the parameter $\theta =(\mu_{1},\sigma_{1},\mu_{2},\sigma_{2},\ldots ,\mu_{n},\sigma_{n})$, it can be shown [22] that the metric in the parametric space of $M_{D}$ is equal to the product metric:

(21) $$d_{D}\left(\theta_{1},\theta_{2}\right)=\sqrt{\sum_{i=1}^{n}d_{F*}^{2}\left(\left(\mu_{1i},\sigma_{1i}\right),\left(\mu_{2i},\sigma_{2i}\right)\right)},$$

where $d_{F*}$ is the Fisher–Rao distance in the univariate case given by [22]:

(22) $$d_{F*}\left(\left(\mu_{1},\sigma_{1}\right),\left(\mu_{2},\sigma_{2}\right)\right)=\sqrt{2}\log\frac{\left|\left(\frac{\mu_{1}}{\sqrt{2}},\sigma_{1}\right)-\left(\frac{\mu_{2}}{\sqrt{2}},-\sigma_{2}\right)\right|+\left|\left(\frac{\mu_{1}}{\sqrt{2}},\sigma_{1}\right)-\left(\frac{\mu_{2}}{\sqrt{2}},\sigma_{2}\right)\right|}{\left|\left(\frac{\mu_{1}}{\sqrt{2}},\sigma_{1}\right)-\left(\frac{\mu_{2}}{\sqrt{2}},-\sigma_{2}\right)\right|-\left|\left(\frac{\mu_{1}}{\sqrt{2}},\sigma_{1}\right)-\left(\frac{\mu_{2}}{\sqrt{2}},\sigma_{2}\right)\right|}.$$

In this space, a curve $\gamma_{D}\left(t\right)=\left(\gamma_{1}\left(t\right),\ldots ,\gamma_{n}\left(t\right)\right)$ is a geodesic if, and only if, $\gamma_{i}\left(t\right)$ is a geodesic curve in the univariate case, for all i=1,…,n. The geodesic curves in the univariate normal distributions space (upper half plane $R\times R^{+}$) are half-vertical lines and half-ellipses centered at σ=0, with eccentricity $\frac{1}{\sqrt{2}}$ [22].

It is important to note that $M_{D}\subset M$ is not totally geodesic. The submanifold of $M_{D}$ composed only by normal distributions with covariance matrices which are multiples of the identity (round normals) is totally geodesic [22]. In fact, this submanifold of round normals is also contained in the totally geodesic submanifold described next.

#### 2.1.4. The Submanifold $M_{D\mu }$ Where Σ Is Diagonal and μ Is an Eigenvector of Σ

Let $M_{D\mu }$ be the n+1-dimensional submanifold composed by distributions with the mean vector $\mu =\mu_{1}e_{i}$ for some $e_{i}\in \left\{e_{1},\ldots ,e_{n}\right\}$ (the canonical basis of $R^{n}$) and diagonal covariance matrix Σ, and without loss of generality, we shall assume that $e_{i}=e_{1}$. An analytic expression for the distance in $M_{D\mu }$ is:

(23) $$d_{D\mu }^{2}\left(\theta_{1},\theta_{2}\right)=d_{F*}^{2}\left(\left(\mu_{11},\sigma_{11}\right),\left(\mu_{21},\sigma_{21}\right)\right)+\sum_{i=2}^{n}d_{F*}^{2}\left(\left(0,\sigma_{1i}\right),\left(0,\sigma_{2i}\right)\right).$$

We proved in [42] that this submanifold is totally geodesic.

### 2.2. Bounds for the Fisher–Rao in M

As mentioned, a closed form for the Fisher–Rao distance between two general normal distributions is not known. In this subsection, we present some bounds for this distance.

#### 2.2.1. A Lower Bound

Calvo and Oller [43] derived a lower bound for the Fisher–Rao distance through an isometric embedding of the parametric space M into the manifold of the positive definite matrices.

**Proposition** **1.** *[43] Given $\theta_{1}=\left(\mu_{1},\Sigma_{1}\right)$ and $\theta_{2}=\left(\mu_{2},\Sigma_{2}\right)$, let:*

(24) $$S_{i}=\left(\begin{array}{cc} \Sigma_{i}+\mu_{i}\mu_{i}^{t} & \mu_{i}^{t}\\ \mu_{i} & 1 \end{array}\right),$$

*i=1,2. A lower bound for the distance between $\theta_{1}$ and $\theta_{2}$ is:*

(25) $$LB\left(\theta_{1},\theta_{2}\right)=\sqrt{\frac{1}{2}\sum_{i=1}^{n+1}{\left[\log\left(\lambda_{i}\right)\right]}^{2}},$$

*where $\lambda_{i}$, 1≤i≤n+1, are the eigenvalues of $S_{1}^{-1/2}S_{2}S_{1}^{-1/2}$.*

We note that this bound satisfies the distance proprieties in M. In [44], through a similar approach, a lower bound for the Fisher–Rao distance was obtained in the more general space of elliptical distributions, restricted to normal distributions, is the above bound.

#### 2.2.2. The Upper Bound $UB_{1}$

In [45], we proposed an upper bound based on an isometry (8) in the manifold M and on the distance in the non-totally geodesic submanifold $M_{D}$ (21), as follows:

**Proposition** **2.** *[45] The Fisher–Rao distance between two multivariate normal distributions $\theta_{1}=\left(\mu_{1},\Sigma_{1}\right)$ and $\theta_{2}=\left(\mu_{2},\Sigma_{2}\right)$ is upper bounded by,*

(26) $$UB_{1}\left(\theta_{1},\theta_{2}\right)=\sqrt{\sum_{i=1}^{n}d_{F*}^{2}\left(\left(0,1\right),\left(\mu_{i},\lambda_{i}\right)\right)},$$

*where $\lambda_{i}$ are the diagonal terms of the matrix* Λ *given by the eigenvalues of $A=\Sigma_{1}^{-(1/2)}\Sigma_{2}\Sigma_{1}^{-(1/2)}=Q\Lambda Q^{t}$, $\mu_{i}$ are the coordinates of $\mu =Q^{t}\Sigma_{1}^{-(1/2)}\left(\mu_{2}-\mu_{1}\right)$, Q is the orthogonal matrix whose columns are the eigenvectors of A and $d_{F*}$ is the Fisher–Rao distance between univariate normal distributions given in Equation (22).*

#### 2.2.3. The Upper Bounds $UB_{2}$ and $UB_{3}$

Considering the Fisher–Rao distance in the totally geodesic submanifold $M_{D\mu }$ and the triangular inequality, we propose another upper bound [42].

Given $\theta_{1}=\left(\mu_{1},\Sigma_{1}\right)$ and $\theta_{2}=\left(\mu_{2},\Sigma_{2}\right)$, we consider the Fisher–Rao distance between $\theta_{0}=\left(0,I_{n}\right)$ and $\theta_{3}=\left(\mu_{3},\Sigma_{3}\right)$ as in Equation (10). Let $\bar{\theta }=\left(\bar{\mu },\bar{\Sigma }\right)$; by the triangular inequality, it follows that:

(27) $$d_{F}\left(\theta_{0},\theta_{3}\right)\leq d_{F}\left(\theta_{0},\bar{\theta }\right)+d_{F}\left(\bar{\theta },\theta_{3}\right).$$

To calculate this bound, we choose $\bar{\theta }$ appropriately. For $\bar{\mu }=\mu_{3}$, note that $d_{F}\left(\bar{\theta },\theta_{3}\right)=d_{\mu }\left(\bar{\theta },\theta_{3}\right)$. Let *P* be an orthogonal matrix such that $P\mu =(|\mu_{3}|,0,\ldots ,0)$ and $D=\mathrm{diag}(d_{1}^{2},d_{2}^{2},\ldots ,d_{n}^{2})$ a diagonal matrix. We will consider $\bar{\Sigma }=P^{-1}DP^{-t}$ and $\theta_{P}=\left(P\mu ,D\right)$. By the isometry $\psi_{\left(c,Q\right)}$, given in Equation (9), for $Q=P^{-1}$ and c=0, it follows:

(28) $$d_{F}\left(\theta_{0},\bar{\theta }\right)=d_{D\mu }\left(\theta_{0},\theta_{P}\right).$$

Then, combining Inequality (27) and Equation (28), the left side of the equation below is an upper bound for the Fisher–Rao distance between $\theta_{1}$ and $\theta_{2}$,

(29) $$d_{F}\left(\theta_{0},\theta_{3}\right)\leq d_{D\mu }\left(\theta_{0},\theta_{P}\right)+d_{\mu }\left(\bar{\theta },\theta_{3}\right).$$

In [42], we derived the upper bound:

(30) $$UB_{2}=d_{D\mu }\left(\theta_{0},\theta_{P}\right)+d_{\mu }\left(\bar{\theta },\theta_{3}\right).$$

through a numerical minimization process by considering the diagonal elements of *D* as a vector that minimizes $d_{D\mu }\left(\theta_{0},\theta_{P}\right)+d_{\mu }\left(\bar{\theta },\theta_{3}\right)$,

(31) $$\left({\bar{d}}_{1},{\bar{d}}_{2},\ldots ,{\bar{d}}_{n}\right)=\min_{\left(d_{1},d_{2},\ldots ,d_{n}\right)}\left\{d_{D\mu }\left(\theta_{0},\theta_{P}\right)+d_{\mu }\left(\bar{\theta },\theta_{3}\right)\right\}.$$

We also derive an analytic upper bound $UB_{3}$ by minimizing of the distance $d_{D\mu }\left(\theta_{0},\theta_{P}\right)$. By expressing this distance in terms of the parameters $\left(\theta_{0},\theta_{P}\right)=\left(\left(0,I_{n}\right),\left(P\mu ,D\right)\right)$, we can show that it reaches the minimum at:

(32) $$D=\mathrm{diag}\left(\sqrt{\frac{|\mu_{3}|+2}{2}},1,\cdots ,1\right).$$

The lower bound of Section 2.2.1 and the upper bounds of Section 2.2.2 and Section 2.2.3 are summarized in Table 1.

Upper and lower bounds have been used to estimate the Fisher–Rao distance in applications such as [37].

#### 2.2.4. Comparisons of the Bounds

In this section, as in [42], we illustrate comparisons between the bounds presented previously.

We consider the bivariate normal distributions model (n=2) and distributions $\theta_{0}$ and $\hat{\theta }=\left(\hat{\mu },\hat{\Sigma }\right)$, where:

(33) $$\hat{\theta }=\left(\hat{\mu },\hat{\Sigma }\right)=\left(\left(\begin{array}{c} \mu \\ 0 \end{array}\right),\left(\begin{array}{cc} \cos(\alpha ) & \sin(\alpha )\\ -\sin(\alpha ) & \cos(\alpha ) \end{array}\right)\left(\begin{array}{cc} \lambda_{1} & 0\\ 0 & \lambda_{2} \end{array}\right)\left(\begin{array}{cc} \cos(\alpha ) & -\sin(\alpha )\\ \sin(\alpha ) & \cos(\alpha ) \end{array}\right)\right).$$

From (10), we can see that there always exists an isometry that converts any two pairs of bivariate distributions into a pair of distributions as above.

We present next a comparison between the lower bound “LB” (25), the upper bounds $UB_{1}$ (26), $UB_{2}$ (31), and $UB_{3}$ (32), and the numerical solution given by the geodesic shooting algorithm (GS) [31] in specific situations.

**Figure 1:**
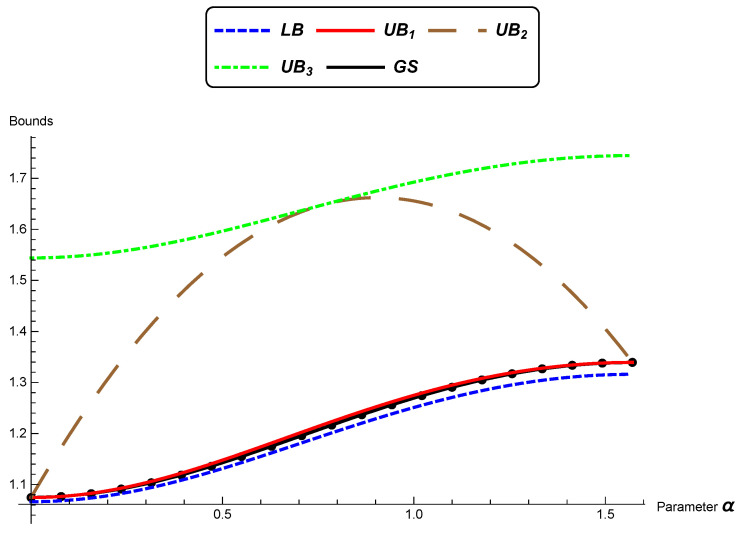
A comparison between the bounds LB, $UB_{1}$, $UB_{2}$, $UB_{3}$, and GS. ($\lambda_{1}=2$, $\lambda_{2}=0.5$, and μ=1 are fixed, and α varies from zero to $\frac{\pi }{2}$).

**Figure 2:**
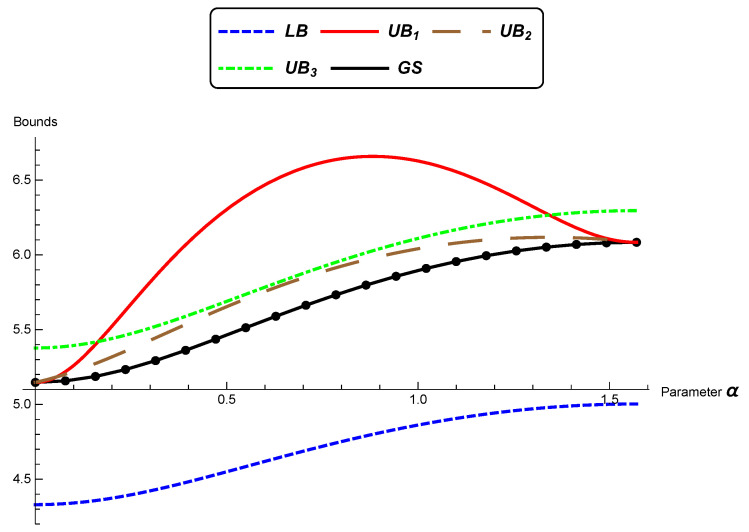
A comparison between the bounds LB, $UB_{1}$, $UB_{2}$, $UB_{3}$, and GS. ($\lambda_{1}=2$, $\lambda_{2}=0.5$, and μ=10 are fixed, and α varies from zero to $\frac{\pi }{2}$).

**Figure 3:**
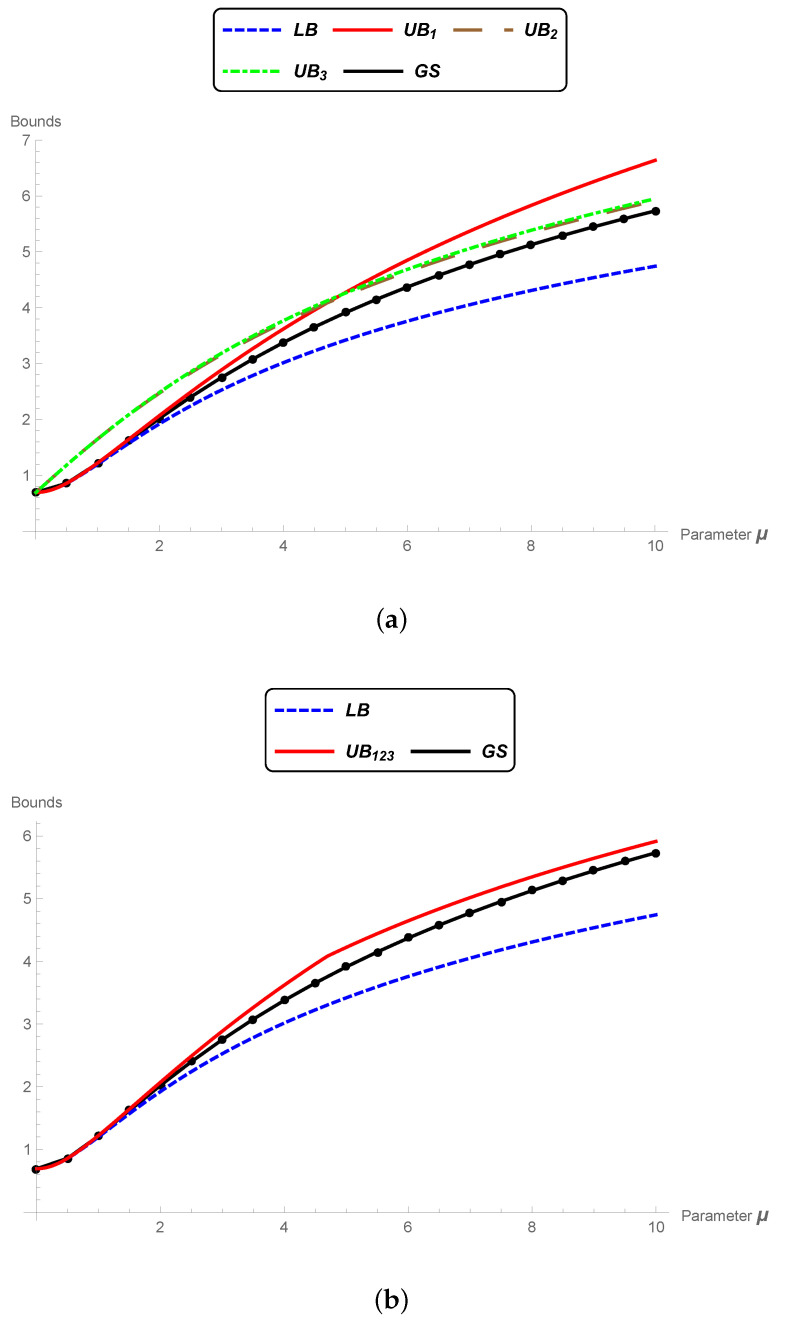
(**a**) A comparison between the bounds LB, $UB_{1}$, $UB_{2}$, $UB_{3}$, and GS. ($\lambda_{1}=2$, $\lambda_{2}=0.5$, and the rotation angle α=π/4 are fixed, and μ varies from zero to 10). (**b**) A comparison between the bounds LB, $UB_{123}$, and GS. ($\lambda_{1}=2$, $\lambda_{2}=0.5$, and the rotation angle α=π/4 are fixed, and μ varies from zero to 10).

In Figure 1, we consider the eigenvalues $\lambda_{1}=2$, $\lambda_{2}=0.5$, and μ=1 to be fixed and α varying from zero to $\frac{\pi }{2}$. We note that the upper bound $UB_{1}$ is very near the lower bound LB and to the numerical solution GS. The other upper bounds are bigger than the bound $UB_{1}$. In Figure 2, it is considered μ=10 and the previous eigenvalues. Now, the best performance is of bounds $UB_{2}$ and $UB_{3}$, which are similar. In Figure 3a, we again keep the eigenvalues; the rotation angle is fixed $\alpha =\frac{\pi }{4}$; and μ varies from zero to 10. We can see similar performances of $UB_{2}$ and $UB_{3}$, which are better than $UB_{1}$ for larger values of μ.

We may also consider the upper bound:

(34) $$UB_{123}\left(\theta_{1},\theta_{2}\right)=\min\left\{UB_{1}\left(\theta_{1},\theta_{2}\right),UB_{2}\left(\theta_{1},\theta_{2}\right),UB_{3}\left(\theta_{1},\theta_{2}\right)\right\}.$$

Figure 3b displays the comparison between LB, $UB_{123}$, and GS for the same data of the comparison in Figure 3a.

## 3. Fisher–Rao Distance Between Special Distributions

In this section, we describe the Fisher–Rao distance in the full space M between special kinds of distributions.

### 3.1. The Fisher–Rao Distance Between Distributions with Common Covariance Matrices

The Fisher–Rao distance between distributions with common covariance matrices given in Section 2.1.1 was restricted to non-totally geodesic submanifold $M_{\Sigma }$. We show next that using the isometry given in (8) and the distance in the submanifold $M_{D\mu }$, it is possible to find a closed form for the distance between two distributions with the same covariance matrix, in the full manifold M.

**Proposition** **3.** *Given two distributions $\theta_{1}=\left(\mu_{1},\Sigma \right)$ and $\theta_{2}=\left(\mu_{2},\Sigma \right)$ in M, let P be an orthogonal matrix such that $P\left(\mu_{2}-\mu_{1}\right)=\left|\mu_{2}-\mu_{1}\right|e_{1}$, and consider the decomposition $UDU^{t}$ of the matrix $P\Sigma P^{t}$,*

(35) $$P\Sigma P^{t}=UDU^{t},$$

*where U is an upper triangular matrix with all diagonal entries equal to one and D is a diagonal matrix. The Fisher–Rao distance between $\theta_{1}$ and $\theta_{2}$ is given by:*

(36) $$d_{F}\left(\theta_{1},\theta_{2}\right)=d_{D\mu }(\left(0,D\right),(|\mu_{2}-\mu_{1}|e_{1},D)).$$

**Proof.**  By considering the isometries $\psi =\psi_{\left(-P\mu_{1},P\right)}$ and $\hat{\psi }=\psi_{\left(0,U^{-1}\right)}$ and the decomposition given by Equation (35), it follows from Equation (9) that:

(37) $$\begin{array}{cc} d_{F}\left(\theta_{1},\theta_{2}\right) & =d_{F}\left(\psi_{\left(-P\mu_{1},P\right)}\left(\theta_{1}\right),\psi_{\left(-P\mu_{1},P\right)}\left(\theta_{2}\right)\right)\\ \; & =d_{F}\left(\left(P\mu_{1}-P\mu_{1},P\Sigma P^{t}\right),\left(P\mu_{2}-P\mu_{1},P\Sigma P^{t}\right)\right)\\ \; & =d_{F}(\left(0,P\Sigma P^{t}\right),(|\mu_{2}-\mu_{1}|e_{1},P\Sigma P^{t})).\\ \; & =d_{F}(\hat{\psi }\left(0,P\Sigma P^{t}\right),\hat{\psi }\left(\right|\mu_{2}-\mu_{1}|e_{1},P\Sigma P^{t}))\\ \; & =d_{F}(\left(U^{-1}0,U^{-1}P\Sigma P^{t}U^{-t}\right),(|\mu_{2}-\mu_{1}|U^{-1}e_{1},U^{-1}P\Sigma P^{t}U^{-t}))\\ \; & =d_{F}\left(\left(0,D\right),(\right|\mu_{2}-\mu_{1}|e_{1},D)). \end{array}$$

Since the distributions (0,D) and $\left(|\mu_{2}-\mu_{1}|e_{1},D\right)$ belong to the submanifold $M_{D\mu }$, we conclude that:

(38) $$d_{F}\left(\theta_{1},\theta_{2}\right)=d_{D\mu }(\left(0,D\right),(|\mu_{2}-\mu_{1}|e_{1},D)).$$

□

**Example** **1.** *Consider two bivariate normal distributions $\theta_{1}=\left({\left(-1,0\right)}^{t},\Sigma \right)$ and $\theta_{2}=\left({\left(6,3\right)}^{t},\Sigma \right)$ with the same covariance matrix:*

$$\Sigma =\left(\begin{array}{cc} 1.1 & 0.9\\ 0.9 & 1.1 \end{array}\right).$$

**Figure 4:**
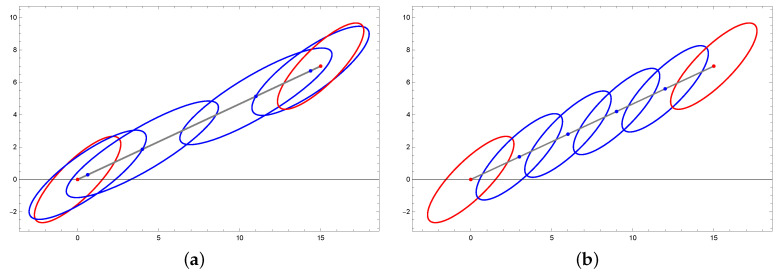
(**a**) Level curves of the distributions in the geodesic curve connecting the bivariate normal distributions $\theta_{1}=\left({\left(-1,0\right)}^{t},\Sigma \right)$ and $\theta_{2}=\left({\left(6,3\right)}^{t},\Sigma \right)$ in M. (**b**) Level curves of the distributions in the geodesic curve connecting the bivariate normal distributions $\theta_{1}=\left({\left(-1,0\right)}^{t},\Sigma \right)$ and $\theta_{2}=\left({\left(6,3\right)}^{t},\Sigma \right)$ in $M_{\Sigma }$.

*Figure 4a illustrates the normal distributions in the geodesic curve connecting connecting $\theta_{1}$ and $\theta_{2}$ in M, and Figure 4b illustrates the geodesic in the submanifold $M_{\Sigma }$. We observe that in M, the shape of the ellipses (contour curves) changes along the path. Furthermore, the Fisher–Rao distance between $\theta_{1}$ and $\theta_{2}$ is $d_{F}\left(\theta_{1},\theta_{2}\right)=5.00648$, which is less than the Mahalanobis distance given in Equation (17), $d_{\Sigma }\left(\theta_{1},\theta_{2}\right)=8.06226$, as expected, since the submanifold $M_{\Sigma }$ is not totally geodesic.*

### 3.2. The Fisher–Rao Distance Between Mirrored Distributions

We consider here two mirrored normal distributions; that is, without loss of generality, if we consider up rotation, the line connecting $\mu_{1}$ and $\mu_{2}$ as parallel to the $e_{1}$-axis, and the covariance matrices $\Sigma_{1}$ and $\Sigma_{2}$ satisfying:

(39) $$\Sigma_{2}=M_{1}\;\Sigma_{1}\;M_{1},\mathrm{where}M_{1}=\left(\begin{array}{cc} -1 & 0\\ 0 & I_{n-1} \end{array}\right).$$

This condition implies also the same eigenvalues for both matrices.

For bivariate normal distributions, we then should have:

(40) $$\theta_{1}=\left(\left(\begin{array}{c} \mu_{1}\\ \mu_{0} \end{array}\right),\left(\begin{array}{cc} \sigma_{11} & \sigma_{12}\\ \sigma_{12} & \sigma_{22} \end{array}\right)\right)\;e\;\theta_{2}=\left(\left(\begin{array}{c} \mu_{2}\\ \mu_{0} \end{array}\right)\left(\begin{array}{cc} \sigma_{11} & -\sigma_{12}\\ -\sigma_{12} & \sigma_{22} \end{array}\right)\right);$$

see Figure 5.

**Figure 6:**
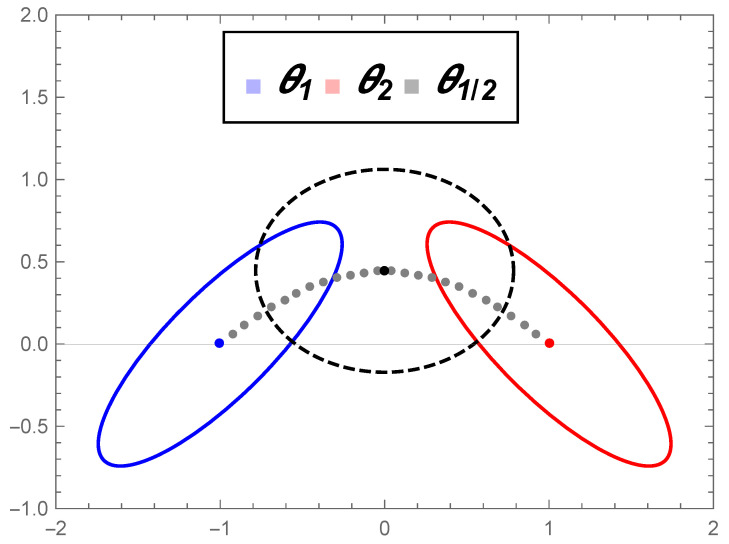
Approximation of the geodesic curve connecting $\theta_{1}$ and $\theta_{2}$ via the geodesic shooting algorithm. The level curve of $\theta_{1/2}$ is the dashed one.

After several experiments using the algorithm *geodesic shooting* for the $\theta_{1}$ and $\theta_{2}$, we have observed that for t=0 the geodesic curve connecting these distributions (γ(t)=(μ(t),Σ(t)), with $\gamma \left(-1\right)=\theta_{1}$ and $\gamma \left(1\right)=\theta_{2}$), satisfies

(41) $$\gamma \left(0\right)\approx \theta_{1/2}=\left(\mu_{1/2},\Sigma_{1/2}\right)=\left(\left(\begin{array}{c} \frac{\mu_{1}+\mu_{2}}{2}\\ \eta \end{array}\right),\left(\begin{array}{cc} d_{11}^{2} & 0\\ 0 & d_{22}^{2} \end{array}\right)\right),$$

(42) $$\gamma^{\prime }\left(0\right)\approx {\hat{\theta }}_{1/2}=\left({\hat{\mu }}_{1/2},{\hat{\Sigma }}_{1/2}\right)=\left(\left(\begin{array}{c} {\hat{\mu }}_{1}\\ 0 \end{array}\right),\left(\begin{array}{cc} 0 & {\hat{\sigma }}_{12}\\ {\hat{\sigma }}_{12} & 0 \end{array}\right)\right).$$

where η, $d_{11}$, and $d_{22}$ are real values; see Figure 6.

The focus here is the “shape” of these distributions. Note that at t=0, the distribution γ(0) appears as $\theta_{1/2}$, which has a diagonal covariance matrix, and the tangent vector $\gamma^{\prime }\left(0\right)$ appears as ${\hat{\theta }}_{1/2}$, which is composed by a mean vector with the second entry equal to zero and by a symmetric covariance matrix with a null diagonal.

This observation inspired us to get an explicit expression for the geodesic connecting two mirrored distributions. Starting with the bi-dimensional case again, we will prove that in fact we have equality in Expressions (41) and (42).

Let γ(t)=(μ(t),Σ(t)), −1≤t≤1, and the geodesic curve in M connecting $\theta_{1}$ and $\theta_{2}$, and consider that $\gamma \left(0\right)=\theta_{1/2}$ and $\gamma^{\prime }\left(0\right)={\hat{\theta }}_{1/2}$. Given the isometry $\psi =\psi_{\left(-\Sigma_{1/2}^{-1/2}\mu_{1/2},\Sigma_{1/2}^{-1/2}\right)}$, we define:

(43) $$\bar{\gamma }\left(t\right)=\left(\bar{\mu }\left(t\right),\bar{\Sigma }\left(t\right)\right):=\psi \left(\gamma \left(t\right)\right)=\left(\Sigma_{1/2}^{-1/2}\left(\mu \left(t\right)-\mu_{1/2}\right),\Sigma_{1/2}^{-1/2}\;\Sigma \left(t\right)\;\Sigma_{1/2}^{-1/2}\right).$$

Then:

(44) $${\bar{\gamma }}^{\prime }\left(t\right)=\left(\frac{d\bar{\mu }\left(t\right)}{dt},\frac{\bar{\Sigma }\left(t\right)}{dt}\right)=\left(\Sigma_{1/2}^{-1/2}\left(\frac{d\mu (t)}{dt}\right),\Sigma_{1/2}^{-1/2}\left(\frac{\Sigma (t)}{dt}\right)\Sigma_{1/2}^{-1/2}\right),$$

(45) $$\begin{array}{cc} \bar{\gamma }\left(0\right)= & \left(\Sigma_{1/2}^{-1/2}\left(\mu_{1/2}-\mu_{1/2}\right),\Sigma_{1/2}^{-1/2}\;\Sigma_{1/2}\;\Sigma_{1/2}^{-1/2}\right)\\ = & \left(0,I_{2}\right)=:\theta_{0} \end{array}$$

and:

(46) $$\begin{array}{cc} {\bar{\gamma }}^{\prime }\left(0\right) & =\left(\Sigma_{1/2}^{-1/2}\;\mu_{1/2}^{\prime },\Sigma_{1/2}^{-1/2}\;\Sigma_{1/2}^{\prime }\;\Sigma_{1/2}^{-1/2}\right)\\ \; & =\left(\left(\begin{array}{c} \frac{\mu^{\prime }\left(0\right)}{d_{11}}\\ 0 \end{array}\right),\left(\begin{array}{cc} 0 & \frac{\sigma_{12}^{\prime }\left(0\right)}{d_{11}d_{22}}\\ \frac{\sigma_{12}^{\prime }\left(0\right)}{d_{11}d_{22}} & 0 \end{array}\right)\right). \end{array}$$

Applying the natural changing of parameters:

(47) $$\left(\delta \left(t\right),\Delta \left(t\right)\right)=\varphi \left(\bar{\mu }\left(t\right),\bar{\Sigma }\left(t\right)\right)=\left(\bar{\Sigma }{\left(t\right)}^{-1}\bar{\mu }\left(t\right),\bar{\Sigma }{\left(t\right)}^{-1}\right),$$

it follows that:

(48) $$\left\{\begin{array}{cc} \frac{d\Delta }{dt}\left(t\right)= & -\Delta \left(t\right)\left(\frac{d\bar{\Sigma }}{dt}\left(t\right)\right)\Delta \left(t\right)\\ \frac{d\delta }{dt}\left(t\right)= & \left(\frac{d\Delta }{dt}\left(t\right)\right)\bar{\mu }\left(t\right)+\Delta \left(t\right)\left(\frac{d\bar{\mu }}{dt}\left(t\right)\right) \end{array}\right..$$

Then, given that $\left(\delta \left(0\right),\Delta \left(0\right)\right)=\left(\bar{\mu }\left(0\right),\bar{\Sigma }\left(0\right)\right)=\left(0,I_{2}\right)$,

(49) $$\left\{\begin{array}{cc} \frac{d\Delta }{dt}\left(0\right)= & -\Delta \left(0\right)\left(\frac{d\bar{\Sigma }}{dt}\left(0\right)\right)\Delta \left(0\right)=-\frac{d\bar{\Sigma }}{dt}\left(0\right)\\ \frac{d\delta }{dt}\left(0\right)= & \left(\frac{d\Delta }{dt}\left(0\right)\right)\bar{\mu }\left(0\right)+\Delta \left(0\right)\left(\frac{d\bar{\mu }}{dt}\left(0\right)\right)=\frac{d\bar{\mu }}{dt}\left(0\right) \end{array}\right..$$

That is, at t=0, the tangent vector $\left(\frac{d\delta }{dt}\left(0\right),\frac{d\Delta }{dt}\left(0\right)\right)$ is equal to the tangent vector in (46). Furthermore, the distributions $\vartheta_{1}=\varphi \left({\bar{\theta }}_{1}\right)=\left({\bar{\Sigma }}_{1}^{-1}{\bar{\mu }}_{1},{\bar{\Sigma }}_{1}^{-1}\right)$ and $\vartheta_{2}=\varphi \left({\bar{\theta }}_{2}\right)=\left({\bar{\Sigma }}_{2}^{-1}{\bar{\mu }}_{2},{\bar{\Sigma }}_{2}^{-1}\right)$ are also mirrored ${\bar{\Sigma }}_{2}^{-1}=M_{1}\;{\bar{\Sigma }}_{1}^{-1}\;M_{1}$. In fact,

(50) $$\begin{array}{cc} \vartheta_{1}= & \left({\bar{\Sigma }}_{1}^{-1}{\bar{\mu }}_{1},{\bar{\Sigma }}_{1}^{-1}\right)\\ = & \left({\left(\Sigma_{1/2}^{-1/2}\;\Sigma_{1}\;\Sigma_{1/2}^{-1/2}\right)}^{-1}\Sigma_{1/2}^{-1/2}\left(\mu_{1}-\mu_{1/2}\right),{\left(\Sigma_{1/2}^{-1/2}\;\Sigma_{1}\;\Sigma_{1/2}^{-1/2}\right)}^{-1}\right)\\ = & \left(\frac{1}{\det(\Sigma_{1})}\left(\begin{array}{c} \sigma_{22}d_{11}\frac{\mu_{1}-\mu_{2}}{2}-\sigma_{12}d_{11}\left(\mu_{0}-\eta \right)\\ \sigma_{11}d_{22}\left(\mu_{0}-\eta \right)-\sigma_{12}d_{22}\frac{\mu_{1}-\mu_{2}}{2} \end{array}\right),\frac{1}{\det(\Sigma_{1})}\left(\begin{array}{cc} \sigma_{22}d_{11}^{2} & -\sigma_{12}d_{11}d_{22}\\ -\sigma_{12}d_{11}d_{22} & \sigma_{11}d_{22}^{2} \end{array}\right)\right), \end{array}$$

and by similar arguments, we obtain:

(51) $$\begin{array}{c} \vartheta_{2}=\left(\frac{1}{\det(\Sigma_{1})}\left(\begin{array}{c} \sigma_{22}d_{11}\frac{\mu_{2}-\mu_{1}}{2}+\sigma_{12}d_{11}\left(\mu_{0}-\eta \right)\\ \sigma_{11}d_{22}\left(\mu_{0}-\eta \right)-\sigma_{12}d_{22}\frac{\mu_{1}-\mu_{2}}{2} \end{array}\right),\frac{1}{\det(\Sigma_{1})}\left(\begin{array}{cc} \sigma_{22}d_{11}^{2} & \sigma_{12}d_{11}d_{22}\\ \sigma_{12}d_{11}d_{22} & \sigma_{11}d_{22}^{2} \end{array}\right)\right). \end{array}$$

**Figure 7:**
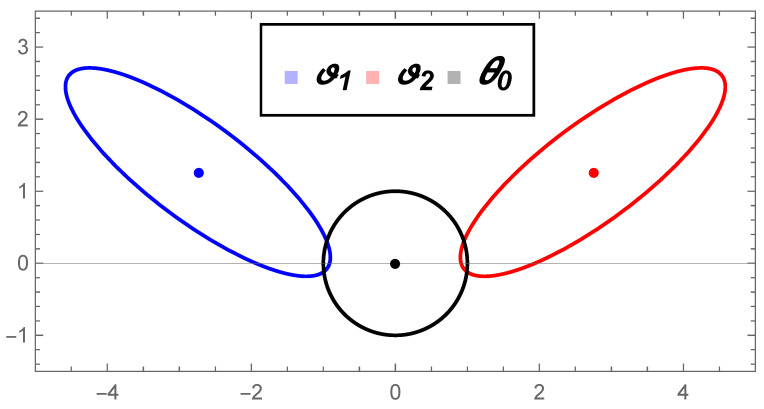
Contour curves of distributions $\vartheta_{1}=\varphi \left({\bar{\theta }}_{1}\right)$ and $\vartheta_{2}=\varphi \left({\bar{\theta }}_{2}\right)$.

Figure 7 illustrates the distributions $\theta_{0}$, $\vartheta_{1}$, and $\vartheta_{2}$.

Conversely, by considering:

(52) $$\left(x,B\right)=\left(\left(\begin{array}{c} x\\ 0 \end{array}\right),\left(\begin{array}{cc} 0 & b\\ b & 0 \end{array}\right)\right)$$

in the initial value problem given in Equations (13) and (14), it follows that the matrix $G^{2}=B^{2}+xx^{t}$ is diagonal. Therefore, the geodesic curve (δ(t),Δ(t)) with initial value $\left(\delta \left(0\right),\Delta \left(0\right)\right)=\theta_{0}$ and tangent vector (x,B) given in Equation (15) can be simplified as follows:

(53) $$\left\{\begin{array}{cc} \delta (t)= & \left(\begin{array}{c} \frac{x\sinh(t\sqrt{b^{2}+2x^{2}})}{\sqrt{b^{2}+2x^{2}}}\\ -\frac{bx(\cosh\left(t\sqrt{b^{2}+2x^{2}}\right)-1)}{b^{2}+2x^{2}} \end{array}\right)\\ \Delta (t)= & \left(\begin{array}{cc} \frac{1}{2}\left(\cosh\left(bt\right)+\cosh\left(t\sqrt{b^{2}+2x^{2}}\right)\right) & -\frac{1}{2}\left(\sinh\left(bt\right)+\frac{b\sinh(t\sqrt{b^{2}+2x^{2}})}{\sqrt{b^{2}+2x^{2}}}\right)\\ -\frac{1}{2}\left(\sinh\left(bt\right)+\frac{b\sinh(t\sqrt{b^{2}+2x^{2}})}{\sqrt{b^{2}+2x^{2}}}\right) & \frac{1}{2}\left(\cosh\left(bt\right)+\frac{2x^{2}+b^{2}\cosh\left(t\sqrt{b^{2}+2x^{2}}\right)}{b^{2}+2x^{2}}\right) \end{array}\right). \end{array}\right.$$

From the parity of the functions sinh(t) and cosh(t), it is possible to show that, given $t_{0}\in R$, the distributions:

$$\left(\delta \left(-t_{0}\right),\Delta \left(-t_{0}\right)\right)\;\mathrm{and}\;\left(\delta \left(t_{0}\right),\Delta \left(t_{0}\right)\right)$$

are also mirrored.

By the above discussion, we conclude that it is possible to calculate the geodesic curve connecting $\theta_{1}$ and $\theta_{2}$ making $\psi^{-1}\left(\varphi^{-1}\left(\delta \left(-1\right),\Delta \left(-1\right)\right)\right)=\theta_{1}$ and $\psi -1\left(\varphi^{-1}\left(\delta \left(1\right),\Delta \left(1\right)\right)\right)=\theta_{2}$. That is, we need to find the values of η, $d_{11}$, and $d_{22}$ of the isometry ψ and the values of (x,B) such that:

(54) $$\left\{\begin{array}{cc} \varphi (\psi \left(\theta_{1}\right))= & \left(\delta (-1),\Delta (-1)\right)\\ \varphi (\psi \left(\theta_{2}\right))= & \left(\delta (1),\Delta (1)\right) \end{array}\right..$$

Since the two equations above are equivalent, it is enough to solve the equation:

(55) $$\left(\delta \left(1\right),\Delta \left(1\right)\right)=\varphi \left(\psi \left(\mu_{2},\Sigma_{2}\right)\right).$$

This is equivalent to solving the system:

(56) $$\left\{\begin{array}{cc} \; & \left(\begin{array}{cc} \frac{1}{d_{11}} & 0\\ 0 & \frac{1}{d_{22}} \end{array}\right)\Delta \left(1\right)\left(\begin{array}{cc} \frac{1}{d_{11}} & 0\\ 0 & \frac{1}{d_{22}} \end{array}\right)=\Delta_{2}\\ \; & \left(\begin{array}{cc} \frac{1}{d_{11}} & 0\\ 0 & \frac{1}{d_{22}} \end{array}\right)\delta \left(1\right)+\Delta_{2}\left(\begin{array}{c} \frac{\mu_{1}+\mu_{2}}{2}\\ \eta \end{array}\right)=\delta_{2} \end{array}\right.,$$

where $\left(\delta_{2},\Delta_{2}\right)=\varphi \left(\mu_{2},\Sigma_{2}\right)$.

The above non-linear system has five equations and five variables $\left(d_{11},d_{22},\eta ,x,b\right)$ and can be solved by an iterative method. With the solution of this system, we can determine the geodesic curve connecting the distributions $\theta_{1}$ and $\theta_{2}$. Moreover, by Equation (16), the Fisher–Rao distance is:

(57) $$d_{F}\left(\theta_{1},\theta_{2}\right)=2d_{F}\left(\theta_{0},\theta_{2}\right)=2d_{F}\left(\left(0,I_{n}\right),\left(\delta \left(1\right),\Delta \left(1\right)\right)\right)=2\sqrt{\frac{1}{2}\mathrm{tr}\left(B^{2}\right)+x^{t}x}=2\sqrt{b^{2}+x^{2}}.$$

We also remark that the curve of the means δ(t) (and therefore, μ(t)) satisfies the equation of a hyperbola; in fact:

(58) $$\frac{{\left(-\frac{bx(\cosh\left(t\sqrt{b^{2}+2x^{2}}\right)-1)}{b^{2}+2x^{2}}-\frac{bx}{b^{2}+2x^{2}}\right)}^{2}}{{\left(\frac{bx}{b^{2}+2x^{2}}\right)}^{2}}-\frac{{\left(\frac{x\sinh(t\sqrt{b^{2}+2x^{2}})}{\sqrt{b^{2}+2x^{2}}}\right)}^{2}}{{\left(\frac{x}{\sqrt{b^{2}+2x^{2}}}\right)}^{2}}=1.$$

Summarizing the above discussion, we have:

**Proposition** **4.**

*(i)*  Expression (57) provides a closed form for the Fisher–Rao distance between two mirrored bivariate normal distributions, based on the solutions of the non-linear system (56).
*(ii)*  The plane curve given by the coordinates of the mean vector in the geodesic connecting two of these distributions is a hyperbola.

Table 2 shows a time comparison between the numerical method proposed here and the geodesic shooting to obtain the Fisher–Rao distance. The distributions used in this experiment were:

(59) $$\theta_{1}=\left(\left(\begin{array}{c} -\mu \\ 0 \end{array}\right),\left(\begin{array}{cc} 0.55 & -0.45\\ -0.45 & 0.55 \end{array}\right)\right)\;\mathrm{and}\;\theta_{2}=\left(\left(\begin{array}{c} \mu \\ 0 \end{array}\right)\left(\begin{array}{cc} 0.55 & 0.45\\ 0.45 & 0.55 \end{array}\right)\right).$$

for different values of μ.

The method proposed here uses a non-linear system for the calculus of the Fisher–Rao distance, so it is faster the geodesic shooting algorithm. Furthermore, we remark that for μ≥7, the geodesic shooting requires additional adaptation to convergence.

Next, we generalize the results of Proposition 4 to pairs of general multivariate normal mirrored distributions. Without loss of generality, we may assume:

(60) $$\theta_{1}=\left(\mu_{1}e_{1},\Sigma_{1}\right)\;\mathrm{and}\;\theta_{2}=\left(\mu_{2}e_{1},\Sigma_{2}\right),$$

with $\Sigma_{2}=M_{1}\;\Sigma_{1}\;M_{1}$ as in (39), that is:

$$\Sigma_{2}=\left\{\begin{array}{ccc} {\hat{\sigma }}_{1j}= & {\hat{\sigma }}_{j1}=-\sigma_{1j},\;j=2,\ldots ,n\\ {\hat{\sigma }}_{ij}= & \sigma_{ij}, & \;\mathrm{otherwise}. \end{array}\right..$$

**Proposition** **5.** *The Fisher–Rao distance between a pair of multivariate mirrored normal distributions $\theta_{1}$ and $\theta_{2}$ (65) is:*

(61) $$d_{F}\left(\theta_{1},\theta_{2}\right)=2\sqrt{\sum_{l=1}^{n-1}b_{l}^{2}+x^{2}},$$

*where:*

(62) $$\left(x,B\right)=\left(\left(\begin{array}{c} x\\ 0\\ \vdots \\ 0 \end{array}\right),\left(\begin{array}{cccc} 0 & b_{1} & \cdots & b_{n-1}\\ b_{1} & 0 & \cdots & 0\\ \vdots & \vdots & \ddots & \vdots \\ b_{n-1} & 0 & \cdots & 0 \end{array}\right)\right).$$

*The values x and $b_{l}$, the non-zero entries of (x,B), are obtained by the solution of the $\left(n+\frac{n(n+1)}{2}\right)$ order non-linear system:*

(63) $$\left\{\begin{array}{c} L^{-1}\Delta \left(1\right)L^{-1}=\Delta_{2}\\ L^{-1}\delta \left(1\right)+\Delta_{2}\mu_{1/2}=\delta_{2} \end{array}\right.,$$

*where $\mu_{1/2}={\left(\frac{\mu_{1}+\mu_{2}}{2},\eta_{1},\ldots ,\eta_{n-1}\right)}^{t}$, L is the Cholesky factor of the matrix $\Sigma_{1/2}=\left(\begin{array}{cc} d_{11} & 0^{t}\\ 0 & D \end{array}\right)$, with D a symmetric (n−1) order matrix, $\left(\delta_{2},\Delta_{2}\right)=\varphi \left(\mu_{2},\Sigma_{2}\right)$, and (δ(t),Δ(t)) is the geodesic curve with initial value $\left(\delta \left(0\right),\Delta \left(0\right)\right)=\theta_{0}$ and tangent vector (x,B) given in Equation (15).*

Let γ(t)=(μ(t),Σ(t)), −1≤t≤1, be the geodesic curve in M connecting $\theta_{1}$ and $\theta_{2}$. The proof is similar to the bivariate case, by considering $\gamma \left(0\right)=\left(\mu_{1/2},\Sigma_{1/2}\right)$,

(64) $$\gamma^{\prime }\left(0\right)={\hat{\theta }}_{1/2}=\left({\hat{\mu }}_{1/2},{\hat{\Sigma }}_{1/2})\right)=\left(\left(\begin{array}{c} \hat{\mu }\\ 0\\ \vdots \\ 0 \end{array}\right),\left(\begin{array}{cccc} 0 & {\hat{\sigma }}_{12} & \cdots & {\hat{\sigma }}_{1n}\\ {\hat{\sigma }}_{12} & 0 & \cdots & 0\\ \vdots & \vdots & \ddots & \vdots \\ {\hat{\sigma }}_{1n} & 0 & \cdots & 0 \end{array}\right)\right)$$

and $\bar{\gamma }\left(t\right)=\psi \left(\gamma \left(t\right)\right)$ where $\psi =\psi_{\left(-L^{-1}\mu_{1/2}L^{-1}\right)}$.

(65) $$\theta_{1}=\left(\mu_{1}e_{1},\Sigma_{1}\right)\;\mathrm{and}\;\theta_{2}=\left(\mu_{2}e_{1},\Sigma_{2}\right),$$

with $\Sigma_{2}=M_{1}\;\Sigma_{1}\;M_{1}$.

Table 3 collects the results in Section 2.1 and the new results of this section.

## 4. Hierarchical Clustering for Diagonal Gaussian Mixture Simplification

A parameterized Gaussian mixture model *f* is a weighted sum of *m* multivariate normal distributions, that is,

$$f\left(x\right)=\sum_{i=1}^{m}w_{i}p_{i}\left(x;\mu_{i},\Sigma_{i}\right),$$

where $x\in R^{n}$, $p_{i}\left(x;\mu_{i},\Sigma_{i}\right)$, i=1,⋯,m, are normal distributions and $w_{i}$, i=1,⋯,m, are mixture, $\sum_{i=1}^{m}w_{i}=1$. In this paper, we call the diagonal Gaussian mixture model (DGMM) the mixture composed only by distributions with diagonal covariance matrices.

Gaussian mixture models (GMM) are used in modeling datasets: image processing, signal processing, and density estimation problems [46,47,48]. In many applications involving mixture models, the computational requirements are of a very high level due to the large number of mixture components. This can be handled if we reduce the number of components of the mixture: given a mixture *f* of *m* components, we want to find a mixture *g* of *l* components, 1≤l<m, such that *g* is a good approximation of *f* with respect to a similarity measure [49]. Gaussian mixture simplification was considered in statistical inference in [50] and to decode low-density lattice codes [51].

In [49] was proposed a hierarchical clustering algorithm to simplify an exponential family mixture model based on Bregman divergences. This section describes an agglomerative hierarchical clustering method based on the Fisher–Rao distance in the submanifold $M_{D}$ (21) to simplify DGMM, and we present an application to image segmentation, complementing what was developed in [52]. We start by introducing the concept of the centroid for a set of distributions in $M_{D}$.

### 4.1. Centroids in the Submanifold $M_{D}$

In [53], Galperin described centroids in the two-dimensional Minkowski model, which can be translated also to the Klein disk and Poincare half-plane models. Given a set of points $q_{i}=\left(x_{q_{i}},y_{q_{i}},z_{q_{i}}\right)$ in the Minkowski model, with associated weights $u_{i}$, the centroid is computed and normalized as:

(66) $$c^{\prime }=\sum_{i}u_{i}q_{i}\;\mathrm{and}\;c=\frac{c^{\prime }}{-x_{c^{\prime }}^{2}-y_{c^{\prime }}^{2}+z_{c^{\prime }}^{2}}.$$

To calculate the centroid c of a subset of points $C=\{\left(w_{i},\theta_{i}\right)\}$, $\theta_{i}=\left(\mu_{i},\sigma_{i}\right)$, the isometries presented in [25] and the relation between the media × standard deviation plane of parameters of univariate normal distributions and the Poincare half-plane given in [22] are used.

Given a dataset $C=\{\left(w_{i},\theta_{i}\right)\}$, where $\theta_{i}=\left(\mu_{1i},\sigma_{1i},\cdots ,\mu_{ni},\sigma_{ni}\right)$ are distributions in $M_{D}$, the centroid of C is:

(67) $$c:=(c_{1},\cdots ,c_{n}),$$

where $c_{j}$, j=1,⋯,n, is the centroid of $C_{j}=\left\{\left(w_{j},\left(\mu_{ji},\sigma_{ji}\right)\right)\right\}$ given in Equation (66).

### 4.2. Hierarchical Clustering Algorithm

Let a DGMM *f* with parameters $C=\{\left(w_{1},\theta_{1}\right),\cdots ,\left(w_{m},\theta_{m}\right)\}$.

In order to apply the hierarchical clustering algorithm, we need to consider the distance between two subsets *A* and *B*. The three most common distances are called linkage criteria and are given by [54]:

- Single linkage:
  (68) $$D\left(A,B\right)=\min\{d_{D}\left(a,b\right);a\in A,b\in B\};$$
- Complete linkage:
  (69) $$D\left(A,B\right)=\max\{d_{D}\left(a,b\right);a\in A,b\in B\};$$
- Group average linkage:
  (70) $$D\left(A,B\right)=\frac{1}{\left|A||B\right|}\sum_{a\in A}\sum_{b\in B}d_{D}\left(a,b\right),$$
  where $d_{D}$ is the distance in the submanifold $M_{D}$ and |X| is the number of elements of a set *X*.

A summary of the hierarchical clustering algorithm (Algorithm 1) [49] using one of these distances is given next.   
**Algorithm 1:** Hierarchical Clustering Algorithm
1:Form *m* clusters $C_{j}=\left\{\left(w_{j},\theta_{j}\right)\right\}$ with one element.2:Find the two closest clusters, $C_{i}$ and $C_{j}$, with respect to a distance *D*, and merge them into asingle cluster $C_{i}\cup C_{j}$.3:Compute distances between the new cluster and each of the old clusters.4:Repeat Steps 2 and 3 until all items are clustered into a single cluster of size *n*.


The simplified DGMM:

$$g=\sum_{j=1}^{l}\beta_{j}g_{j}$$

of *l* components is built from the *l* subsets $C_{1}$, …, $C_{l}$ remaining after the iteration n−l of the hierarchical clustering algorithm. In this work, we choose the parameters of $g_{j}$ in two ways: as the centroid in the submanifold $M_{D}$ (Fisher–Rao hierarchical clustering) and as the Bregman left-sided centroid [49] (Bregman–Fisher–Rao hierarchical clustering) of the subset $C_{j}$ with weights $\beta_{j}=\sum_{\left(w_{i},\theta_{i}\right)\in C_{j}}w_{i}$.

As remarked in [49], the hierarchical clustering algorithm allows introducing a method to learn the optimal number of components in the simplified mixture *g*. Thus, *g* must be as compact as possible and reach a minimum prescribed quality $d_{KL}\left(f||g\right)\leq \tau$, where $d_{KL}\left(f||g\right)$ is the Kullback–Leibler divergence.

### 4.3. Experiments in Image Segmentation

We can apply the Fisher–Rao and the Bregman–Fisher–Rao hierarchical clusterings to simplify a mixture of exponential families in the context of clustering-based image segmentation as was done in [49] for the Bregman hierarchical clustering. Given an input color image *I*, we adapt the Bregman soft clustering algorithm to generate a DGMM *f* of 32 components, which models the image pixels. We point out that the restriction considered in this paper (only DGMM) is also used in many applications due its much lower computational cost. We consider here a pixel $\rho =(\rho_{R},\rho_{G},\rho_{B})$ as a point in $R^{3}$, where $\rho_{R}$, $\rho_{G}$, and $\rho_{B}$ are the RGB color information. For image segmentation, we can say that the image pixel ρ belongs to the class $C_{j}$ when:

$$p_{j}\left(\rho ;\mu_{j},\Sigma_{j}\right)>p_{i}\left(\rho ;\mu_{i},\Sigma_{i}\right),\;\forall i\in \left\{1,\cdots ,m\right\}\setminus \left\{j\right\}.$$

Thus, the segmented image is illustrated by replacing the color value of the pixel ρ by the mean $\mu_{j}$ of the Gaussian $p_{j}$.

**Figure 8:**
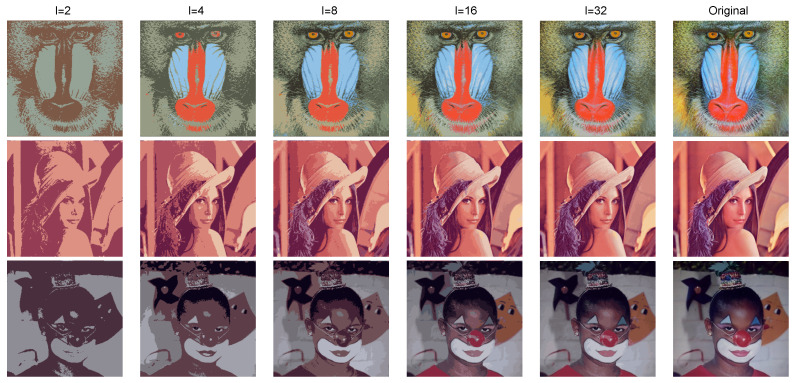
Illustration of the mixture simplification using the Fisher–Rao clustering, where *l* is the number of components of the mixture (the last column is the original figure).

Using the the Fisher–Rao and the Bregman–Fisher–Rao hierarchical clusterings, we simplify the mixture *f* into mixtures *g* of *l* components with l={2,4,8,16}. Each mixture gives one image segmentation. The linkage criterion used here was the complete linkage (68), which has presented better results in our simulations. Figure 8 shows the segmentation of the Baboon, Lena, and Clown input images given by the Bregman–Fisher–Rao hierarchical clustering. The number of colors in each image is equal to the number of components in the simplified mixture *g*.

**Figure 9:**
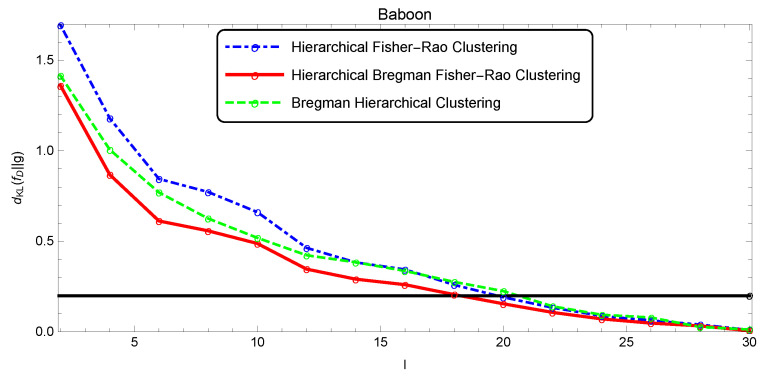
Illustration of the simplification quality of the mixture modeling Baboon image.

**Figure 10:**
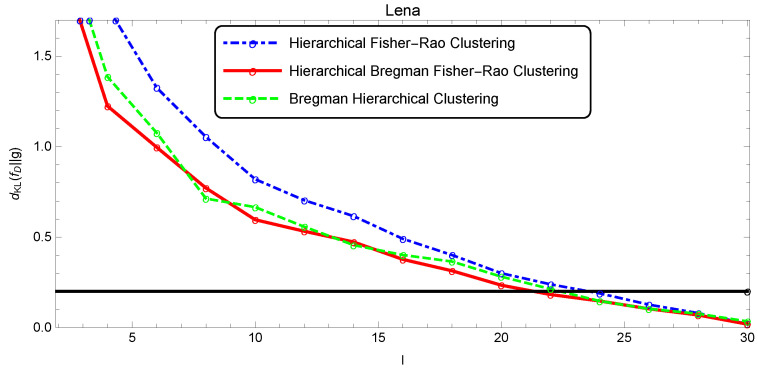
Illustration of the simplification quality of the mixture modeling Lena image.

**Figure 11:**
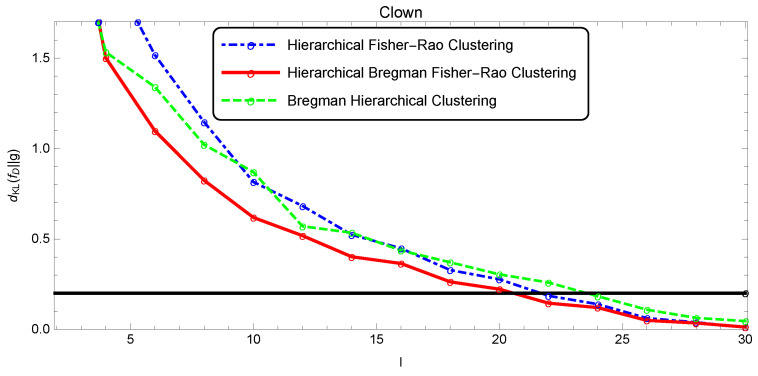
Illustration of the simplification quality of the mixture modeling Clown image.

The quality of the segmentation was analyzed as a function of *l* through the Kullback–Leibler divergence estimated by the Monte Carlo method, since there was no closed form for this measure (five thousand points were randomly drawn to estimate $d_{KL}\left(f||g\right)$). Figure 9, Figure 10 and Figure 11 show the evolution of the simplification quality as a function of the number of components *l* for the Baboon, Lena, and Clown images, using the Bregman, the Fisher–Rao, and the Bregman–Fisher–Rao hierarchical clustering algorithms. We observed that the image quality increased ($d_{KL}\left(f||g\right)$ decreased) with *l*, as expected, and the behavior was similar in all clustering algorithms. In general, the Bregman–Fisher–Rao hierarchical clustering algorithm presented better results. Considering the constraint τ=0.2, the learning process provided, for the Bregman–Fisher–Rao hierarchical clustering, mixtures of 19, 21, and 21 as optimal simplifications for the images of the Baboon, Lena, and Clown, respectively.

## 5. Concluding Remarks

The Fisher–Rao distance was approached here in the space of multivariate normal distributions. Initially, as in [38], we summarized some known closed forms for this distance in submanifolds of this model and some bounds for the general case. A closed form for the Fisher–Rao distance between distributions with the same covariance matrix was obtained in Proposition 3, and we also have derived a non-linear system characterizing the distance between two distributions with mirrored covariance matrices in Proposition 5. Some perspectives for future research related to this topic include deriving new bounds for the Fisher–Rao distance in the general case, by using these special distributions, to characterize as non-linear systems the distances between other types of distributions and to extend the closed forms and bounds presented here to the space of elliptical distributions. Finally, we have extended the analysis of the Bregman–Fisher–Rao hierarchical clustering algorithm to simplify Gaussian mixtures in the context of clustering-based image segmentation given in [52] with comparative results that encourage the use of the Fisher–Rao distance in other clustering or classification algorithms.

## Figures and Tables

**Figure 5:**
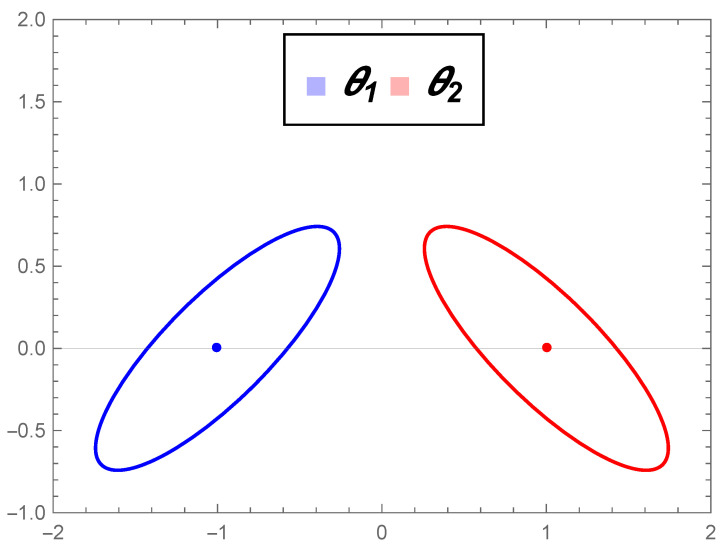
Example of level curves of mirrored distributions where $\theta_{1}$ and $\theta_{2}$ are given by Equation (40).

**Table 1:** The lower bound $LB(\theta_{1},\theta_{2})$ and the upper bounds $UB_{1}\left(\theta_{1},\theta_{2}\right)$, $UB_{2}\left(\theta_{1},\theta_{2}\right)$ and $UB_{3}\left(\theta_{1},\theta_{2}\right)$ for the Fisher–Rao distance, $d_{F}\left(\theta_{1},\theta_{2}\right)$, between distributions $\theta_{1}=\left(\mu_{1},\Sigma_{1}\right)$ and $\theta_{2}=\left(\mu_{2},\Sigma_{2}\right)$ in M. $d_{F*}$ is the distance between univariate normal distributions given in Equation (22).

$LB\left(\theta_{1},\theta_{2}\right)\;=\;\sqrt{\frac{1}{2}\sum_{i=1}^{n+1}{\left[\log\left(\lambda_{i}\right)\right]}^{2}}$	$\begin{array}{ll} – & S_{i}=\left(\begin{array}{cc} \Sigma_{i}+\mu_{i}^{t}\mu_{i} & \mu_{i}^{t}\\ \mu_{i} & 1 \end{array}\right);\\ – & \lambda_{i}\mathrm{are}\mathrm{the}\mathrm{eigenvalues}\mathrm{of}S_{1}^{-1/2}S_{2}S_{1}^{-1/2}. \end{array}$
$UB_{1}\left(\theta_{1},\theta_{2}\right)\;=\;\sqrt{\sum_{i=1}^{n}d_{F*}^{2}\left(\left(0,1\right),\left(\mu_{i},\lambda_{i}\right)\right)}$	$\begin{array}{ll} – & \Sigma_{1}^{-(1/2)}\Sigma_{2}\Sigma_{1}^{-(1/2)}=Q\Lambda Q^{t};\\ – & \lambda_{i}\mathrm{are}\mathrm{the}\mathrm{diagonal}\mathrm{terms}\mathrm{of}\mathrm{the}\mathrm{matrix}\Lambda ;\\ – & \mu_{i}\mathrm{are}\mathrm{the}\mathrm{coordinates}\mathrm{of}\mu =Q^{t}\Sigma_{1}^{-(1/2)}\left(\mu_{2}-\mu_{1}\right). \end{array}$
$UB_{2}\left(\theta_{1},\theta_{2}\right)\;=\;\sqrt{\frac{1}{2}\sum_{i=1}^{n}{\left[\log\left(\lambda_{i}\right)\right]}^{2}}\;\;+$ $\sqrt{d_{F*}^{2}\left(\left(0,1\right),(\right|\mu_{3}|,{\bar{d}}_{1}))+\sum_{i=2}^{m}d_{F*}^{2}\left(\left(0,1\right),\left(0,{\bar{d}}_{i}\right)\right)}$	$\begin{array}{ll} – & \mu_{3}=\Sigma_{1}^{-(1/2)}(\mu_{2}-\mu_{1});\\ – & {\bar{d}}_{i}\mathrm{is}\mathrm{given}\mathrm{by}\;(31);\\ – & \Sigma_{3}=\Sigma_{1}^{-(1/2)}\Sigma_{2}\Sigma_{1}^{-(1/2)};\\ – & P\;\mathrm{is}\mathrm{an}\mathrm{orthogonal}\mathrm{matrix}\mathrm{such}\mathrm{that}\\ & \;P\mu =(|\mu_{3}|,0,\ldots ,0);\\ – & \bar{\Sigma }=P^{-1}\Sigma_{3}P^{-t};\\ – & \lambda_{i}\mathrm{are}\mathrm{the}\mathrm{eigenvalues}\mathrm{of}{\bar{\Sigma }}^{-(1/2)}\Sigma_{3}{\bar{\Sigma }}^{-(1/2)}. \end{array}$
$UB_{3}\left(\theta_{1},\theta_{2}\right)\;=\;\sqrt{\frac{1}{2}\sum_{i=1}^{n}{\left[\log\left(\lambda_{i}\right)\right]}^{2}}\;\;+$ $d_{F*}\left(\left(0,1\right),\left(|\mu_{3}|,\sqrt{\frac{|\mu_{3}|+2}{2}}\right)\right)$	$\begin{array}{ll} – & \mu_{3}=\Sigma_{1}^{-(1/2)}\left(\mu_{2}-\mu_{1}\right);\\ – & \Sigma_{3}=\Sigma_{1}^{-(1/2)}\Sigma_{2}\Sigma_{1}^{-(1/2)};\\ – & P\;\mathrm{is}\mathrm{an}\mathrm{orthogonal}\mathrm{matrix}\mathrm{such}\mathrm{that}\\ & \;P\mu =(|\mu_{3}|,0,\ldots ,0);\\ – & \bar{\Sigma }=P^{-1}\Sigma_{3}P^{-t};\\ – & \lambda_{i}\mathrm{are}\mathrm{the}\mathrm{eigenvalues}\mathrm{of}{\bar{\Sigma }}^{-(1/2)}\Sigma_{3}{\bar{\Sigma }}^{-(1/2)}. \end{array}$

**Table 2:** A time comparison between the numerical method proposed here and the geodesic shooting to calculate the distance between two mirrored distributions.

μ	$d_{F}\left(\theta_{1},\theta_{2}\right)$	Time Systems (s)	Time G.Shooting (s)
1	2.77395	0.046875	4.70313
2	3.67027	0.046875	5.60938
3	4.52933	0.0625	7.10938
4	5.26093	0.078125	9.17188
5	5.87480	0.046875	12.5313
6	6.39439	0.0625	18.4219
7	6.84043	0.078125	492.563
8	7.22903	0.0625	574.422
9	7.57221	0.046875	917.859
10	7.87896	0.046875	1007.13

**Table 3:** Closed forms for the Fisher–Rao distance in submanifolds of M and the distance in M between pairs of special distributions.

Distance in Non-totally Geodesic Submanifolds	
Submanifold	Distance
$\begin{array}{ccc} M_{\Sigma } & = & \left\{\begin{array}{c} p_{\theta }\;;\;\;\theta =\left(\mu ,\Sigma \right),\\ \Sigma =\Sigma_{0}\in P_{n}\left(R\right)\;\mathrm{constant} \end{array}\right\},\\ \theta_{i} & = & \left(\mu_{i},\Sigma_{0}\right) \end{array}$	$d_{\Sigma }\left(\theta_{1},\theta_{2}\right)\;=\;\sqrt{{\left(\mu_{1}-\mu_{2}\right)}^{t}\Sigma_{0}^{-1}\left(\mu_{1}-\mu_{2}\right)}$
$\begin{array}{ccc} M_{D} & = & \left\{\begin{array}{c} p_{\theta }\;;\;\;\theta =\left(\mu ,\Sigma \right),\\ \Sigma =\mathrm{diag}(\sigma_{1}^{2},\sigma_{2}^{2},\ldots ,\sigma_{n}^{2}) \end{array}\right\},\\ \theta_{i} & = & \left(\mu_{1i},\sigma_{1i},\mu_{2i},\sigma_{2i},\ldots ,\mu_{ni},\sigma_{ni}\right) \end{array}$	$d_{D}\left(\theta_{1},\theta_{2}\right)\;=\;\sqrt{\sum_{i=1}^{n}d_{F}^{2}\left(\left(\mu_{1i},\sigma_{1i}\right),\left(\mu_{2i},\sigma_{2i}\right)\right)}$
**Distance in Totally Geodesic Submanifolds**	
$\begin{array}{ccc} M_{\mu } & = & \left\{\begin{array}{c} p_{\theta }\;;\;\;\theta =\left(\mu ,\Sigma \right),\\ \mu =\mu_{0}\in R^{n}\;\mathrm{contant} \end{array}\right\},\\ \theta_{i} & = & \left(\mu_{0},\Sigma_{i}\right) \end{array}$	$d_{F}\left(\theta_{1},\theta_{2}\right)\;=\;\sqrt{\frac{1}{2}\sum_{i=1}^{n}{\left[\log\left(\lambda_{i}\right)\right]}^{2}}$, where $\lambda_{i}$ are the eigenvalues of $\Sigma_{1}^{-1/2}\Sigma_{2}\Sigma_{1}^{-1/2}$
$\begin{array}{ccc} M_{D\mu } & = & \left\{\begin{array}{c} \{p_{\theta }\;;\;\;\theta =\left(\mu ,\Sigma \right),\\ \mu \;\mathrm{is}\;\mathrm{an}\;\mathrm{eigenvector}\;\mathrm{of}\;\\ \Sigma =\mathrm{diag}(\sigma_{1}^{2},\sigma_{2}^{2},\cdots ,\sigma_{n}^{2})\} \end{array}\right\},\\ \theta_{i} & = & \left(\mu_{1i},\sigma_{1i},\sigma_{2i},\ldots ,\sigma_{ni}\right) \end{array}$	$d_{D\mu }\left(\theta_{1},\theta_{2}\right)\;=\;(d_{F}^{2}\left(\left(\mu_{11},\sigma_{11}\right),\left(\mu_{21},\sigma_{21}\right)\right)\;\;+$ $\;\sum_{i=2}^{n}d_{F}^{2}\left(\left(0,\sigma_{1i}\right),\left(0,\sigma_{2i}\right)\right){)}^{1/2}$
**Distance Between Special Distributions in M**	
Distributions with Common Covariance Matrices, $\theta_{i}\;=\;\left(\mu_{i},\Sigma_{0}\right)$	$d_{F}\left(\theta_{1},\theta_{2}\right)\;=\;d_{D\mu }\left(\left(0,D\right),(|\mu_{2}-\mu_{1}|e_{1},D)\right)$, where *P* is an orthogonal matrix such that $P\left(\mu_{2}-\mu_{1}\right)=\left|\mu_{2}-\mu_{1}\right|e_{1}$ and $P\Sigma P^{t}=UDU^{t}$
Mirrored Distributions, $\theta_{1}\;=\;\left(\mu_{1}e_{1},\Sigma_{1}\right)$ and $\theta_{2}\;=\;\left(\mu_{2}e_{1},\Sigma_{2}\right)$, with $\Sigma_{2}\;=\;M_{1}\;\Sigma_{1}\;M_{1}$	$d_{F}\left(\theta_{1},\theta_{2}\right)\;=\;2\sqrt{\sum_{l=1}^{n-1}b_{l}^{2}+x^{2}}$, where *x* and $b_{i}$ are obtained by the solution of Equation (63)

## References

[1] Calin O., Udriste C. (2014). Geometric Modeling in Probability and Statistics. Mathematics and Statistics.

[2] Nielsen F. (2018). An elementary introduction to information geometry. arXiv.

[3] Amari S., Nagaoka H. (2000). Methods of Information Geometry. Translations of Mathematical Monographs.

[4] Amari S. (2016). Information Geometry and Its Applications.

[5] Ay N., Jost J., Vân Lê H., Schwachhöfer L. (2015). Information geometry and sufficient statistics. Probab. Theory Relat. Fields.

[6] Vân Lê H. (2017). The uniqueness of the Fisher metric as information metric. Ann. Inst. Stat. Math..

[7] Gibilisco P., Riccomagno E., Rogantin M.P., Wynn H.P. (2010). Algebraic and Geometric Methods in Statistics.

[8] Chentsov N.N. (1982). Statistical Decision Rules and Optimal Inference.

[9] Campbell L.L. (1986). An extended Cencov characterization of the information metric. Proc. Am. Math. Soc..

[10] Vân Lê H. (2006). Statistical manifolds are statistical models. J. Geom..

[11] Mahalanobis P.C. (1936). On the generalized distance in statistics. Proc. Natl. Inst. Sci..

[12] Bhattacharyya A. (1943). On a measure of divergence between two statistical populations defined by their probability distributions. Bull. Calcutta Math. Soc..

[13] Hotelling H. (1930). Spaces of statistical parameters. Bull. Am. Math. Soc. (AMS).

[14] Rao C.R. (1945). Information and the accuracy attainable in the estimation of statistical parameters. Bull. Calcutta Math. Soc..

[15] Fisher R.A. (1921). On the mathematical foundations of theoretical statistics. Philos. Trans. R. Soc. Lond..

[16] Burbea J. (1986). Informative geometry of probability spaces. Expo. Math..

[17] Skovgaard L.T. (1984). A Riemannian geometry of the multivariate normal model. Scand. J. Stat..

[18] Atkinson C., Mitchell A.F.S. (1981). Rao’s Distance Measure. Sankhyã Indian J. Stat..

[19] Kullback S., Leibler R.A. (1951). On information and sufficiency. Ann. Math. Stat..

[20] Villani C. (2009). Optimal Transport, Old and New. Grundlehren der Mathematischen Wissenschaften.

[21] Amari S. (1985). Differential Geometrical Methods in Statistics.

[22] Costa S.I.R., Santos S.A., Strapasson J.E. (2015). Fisher information distance: A geometrical reading. Discret. Appl. Math..

[23] Angulo J., Velasco-Forero S. (2014). Morphological processing of univariate Gaussian distribution-valued images based on Poincaré upper-half plane representation. Geometric Theory of Information.

[24] Maybank S.J., Ieng S., Benosman R. (2012). A Fisher–Rao metric for paracatadioptric images of lines. Int. J. Comput. Vis..

[25] Schwander O., Nielsen F. Model centroids for the simplification of kernel density estimators. Proceedings of the 2012 IEEE International Conference on Acoustics, Speech and Signal Processing (ICASSP).

[26] Taylor S. (2019). Clustering Financial Return Distributions Using the Fisher Information Metric. Entropy.

[27] Eriksen P.S. (1986). Geodesics Connected with the Fischer Metric on the Multivariate Normal Manifold.

[28] Calvo M., Oller J.M. (1991). An explicit solution of information geodesic equations for the multivariate normal model. Stat. Decis..

[29] Lenglet C., Rousson M., Deriche R., Faugeras O. (2006). Statistics on the manifold of multivariate normal distributions. Theory and application to diffusion tensor MRI processing. J. Math. Imaging Vis..

[30] Moakher M., Mourad Z. (2011). The Riemannian geometry of the space of positive-definite matrices and its application to the regularization of positive-definite matrix-valued data. J. Math. Imaging Vis..

[31] Han M., Park F.C. (2014). DTI Segmentation and Fiber Tracking Using Metrics on Multivariate Normal Distributions. J. Math. Imaging Vis..

[32] Verdoolaege G., Scheunders P. (2011). Geodesics on the manifold of multivariate generalized Gaussian distributions with an application to multicomponent texture discrimination. Int. J. Comput. Vis..

[33] Tang M., Rong Y., Zhou J., Li X.R. (2018). Information geometric approach to multisensor estimation fusion. IEEE Trans. Signal Process..

[34] Poon C., Keriven N., Peyré G. (2018). Support Localization and the Fisher Metric for off-the-grid Sparse Regularization. arXiv.

[35] Gattone S.A., De Sanctis A., Puechmorel S., Nicol F. (2018). On the geodesic distance in shapes K-means clustering. Entropy.

[36] Gattone S.A., De Sanctis A., Russo T., Pulcini D. (2017). A shape distance based on the Fisher–Rao metric and its application for shapes clustering. Phys. A Stat. Mech. Appl..

[37] Pilté M., Barbaresco F. Tracking quality monitoring based on information geometry and geodesic shooting. Proceedings of the 2016 17th International Radar Symposium (IRS).

[38] Pinele J., Costa S.I., Strapasson J.E. (2019). On the Fisher–Rao Information Metric in the Space of Normal Distributions. International Conference on Geometric Science of Information.

[39] Burbea J., Rao C.R. (1982). Entropy differential metric, distance and divergence measures in probability spaces: A unified approach. J. Multivar. Anal..

[40] Porat B., Benjamin F. (1986). Computation of the exact information matrix of Gaussian time series with stationary random components. IEEE Trans. Acoust. Speech Signal Process..

[41] Siegel C.L. (1943). Symplectic geometry. Am. J. Math..

[42] Strapasson J.E., Pinele J., Costa S.I.R. A totally geodesic submanifold of the multivariate normal distributions and bounds for the Fisher–Rao distance. Proceedings of the IEEE Information Theory Workshop (ITW).

[43] Calvo M., Oller J.M. (1990). A distance between multivariate normal distributions based in an embedding into the Siegel group. J. Multivar. Anal..

[44] Calvo M., Oller J.M. (2002). A distance between elliptical distributions based in an embedding into the Siegel group. J. Comput. Appl. Math..

[45] Strapasson J.E., Porto J., Costa S.I.R. (2015). On bounds for the Fisher–Rao distance between multivariate normal distributions. Aip Conf. Proc..

[46] Zhang K., Kwok J.T. (2010). Simplifying mixture models through function approximation. IEEE Trans. Neural Netw..

[47] Davis J.V., Dhillon I.S. Differential entropic clustering of multivariate gaussians. Proceedings of the 2006 Advances in Neural Information Processing Systems.

[48] Goldberger J., Greenspan H.K., Dreyfuss J. (2008). Simplifying mixture models using the unscented transform. IEEE Trans. Pattern Anal. Mach. Intell..

[49] Garcia V., Nielsen F. (2010). Simplification and hierarchical representations of mixtures of exponential families. Signal Process..

[50] Bar-Shalom Y., Li X. (1993). Estimation and Tracking: Principles, Techniques and Software.

[51] Kurkoski B., Dauwels J. Message-passing decoding of lattices using Gaussian mixtures. Proceedings of the 2008 IEEE International Symposium on Information Theory.

[52] Strapasson J.E., Pinele J., Costa S.I.R. Clustering using the Fisher–Rao distance. Proceedings of the IEEE Sensor Array and Multichannel Signal Processing Workshop.

[53] Galperin G.A. (1993). A concept of the mass center of a system of material points in the constant curvature spaces. Commun. Math. Phys..

[54] Nielsen F. (2016). Introduction to HPC with MPI for Data Science. Undergraduate Topics in Computer Science.

